# Understanding primary care transformation and implications for ageing populations and health inequalities: a systematic scoping review of new models of primary health care in OECD countries and China

**DOI:** 10.1186/s12916-023-03033-z

**Published:** 2023-08-24

**Authors:** D. A. G Henderson, E Donaghy, M Dozier, B Guthrie, H Huang, M Pickersgill, E Stewart, A Thompson, H. H. X Wang, S. W Mercer

**Affiliations:** 1 Centre for Population Health Sciences, Usher Institute, University of Edinburgh, Edinburgh, UK; 2 College of Medicine and Veterinary Medicine, University of Edinburgh, Edinburgh, UK; 3 School of Social Work and Social Policy, University of Strathclyde, Glasgow, UK; 4 School of Social and Political Sciences, University of Edinburgh, Edinburgh, UK; 5 School of Public Health, Sun Yat-Sen University, Guangzhou, China

**Keywords:** Primary care, Ageing, Scoping review, Health inequalities

## Abstract

**Background:**

Many countries have introduced reforms with the aim of primary care transformation (PCT). Common objectives include meeting service delivery challenges associated with ageing populations and health inequalities. To date, there has been little research comparing PCT internationally. Our aim was to examine PCT and new models of primary care by conducting a systematic scoping review of international literature in order to describe major policy changes including key ‘components’, impacts of new models of care, and barriers and facilitators to PCT implementation.

**Methods:**

We undertook a systematic scoping review of international literature on PCT in OECD countries and China (published protocol: https://osf.io/2afym). Ovid [MEDLINE/Embase/Global Health], CINAHL Plus, and Global Index Medicus were searched (01/01/10 to 28/08/21). Two reviewers independently screened the titles and abstracts with data extraction by a single reviewer. A narrative synthesis of findings followed.

**Results:**

A total of 107 studies from 15 countries were included. The most frequently employed component of PCT was the expansion of multidisciplinary teams (MDT) (46% of studies). The most frequently measured outcome was GP views (27%), with  < 20% measuring patient views or satisfaction. Only three studies evaluated the effects of PCT on ageing populations and 34 (32%) on health inequalities with ambiguous results. For the latter, PCT involving increased primary care access showed positive impacts whilst no benefits were reported for other components. Analysis of 41 studies citing barriers or facilitators to PCT implementation identified leadership, change, resources, and targets as key themes.

**Conclusions:**

Countries identified in this review have used a range of approaches to PCT with marked heterogeneity in methods of evaluation and mixed findings on impacts. Only a minority of studies described the impacts of PCT on ageing populations, health inequalities, or from the patient perspective. The facilitators and barriers identified may be useful in planning and evaluating future developments in PCT.

**Supplementary Information:**

The online version contains supplementary material available at 10.1186/s12916-023-03033-z.

## Background

Primary care is an important mechanism for managing health care needs of populations by providing integrated, holistic care with the aim of preventing, delaying, or minimising the impacts of multiple chronic conditions on health [1, 2]. In the absence of dedicated resource to meet the needs of ageing populations and the increasing prevalence of multimorbidity, health care services are under increasing strain [3]. Internationally, guided by the World Health Organization (WHO) [1], this has resulted in the reorganisation of primary care via policy reform with the aim of managing increased demand for services whilst improving efficiency and effectiveness [2], often in the absence of additional substantive investment [4]. A recent World Health Organization report reaffirms that primary care should promote principles of comprehensive integrated health care. It further encourages the delivery of care via multidisciplinary teams (MDT) [5]. The Organisation for Economic Cooperation and Development (OECD) additionally recognises the broader roles that patients should play in the design of primary care [2].

The reorganisation of primary care is often referred to as primary care transformation (PCT). We define this as a collection of policy-driven measures which are combined differently across system contexts. Previous reviews of PCT have focused on only one country [6–8], focused on a limited number of interventions or outcomes [9], searched only a limited number of databases [9], or did not fully describe the papers included in the review [10]. None of the reviews focused on health inequalities or included an appraisal of barriers and facilitators to PCT implementation.

We aimed to review the literature from OECD countries and China on PCT in order to (a) describe major policy-driven changes in primary care systems including key ‘components’; (b) describe the impacts of these new models, particularly in the context of ageing populations and health inequalities; and (c) describe barriers and facilitators to implementation of these new models and primary care transformation more generally.

## Methods

A systematic scoping review [11] was conducted following the Preferred Reporting Items for Systematic Reviews and Meta-Analyses (PRISMA) guidelines (Additional file 1: Table S1) [12]. The review protocol was registered with the Open Science Framework (OSF) registry (https://osf.io/2afym).

We included studies published in English from 2010 onwards in international peer-reviewed journals. Included studies contained primary or secondary quantitative and/or qualitative data on PCT based in OECD member states or China. We included China—an exemplar of a large middle-income country with one of the fastest growing ageing populations—with OECD countries to capture evidence from the wide-ranging 2009 Health Care Reform [13]. We excluded conference proceedings, discussion papers, opinion pieces, editorials, grey literature, policy documents, and clinical guidelines.

Following the Population, Intervention, Comparator, and Outcome (PICO) framework, the primary aim was to include studies covering the whole population (as primary care does). Included interventions were large-scale policy-driven changes implemented at the system level as defined by Best et al. [14]. Of particular interest were studies examining the impacts on older people and/or health inequalities. Given the expected heterogeneity of results, we included all types of study, including a comparator group or not, and any outcome studied.

An electronic search was conducted in MEDLINE, Embase, Global Health, the Cumulative Index of Nursing and Allied Health Literature (CINAHL), and Global Index Medicus. The search strategy was developed with the assistance of an information specialist (MD) and refined by team members (DH, HH, ED and SWM). Search terms focussed on two main areas: primary care and/or clinicians, and transformation or health policy. Full search terms are provided in Additional file 1: Table S2. Final searches were run on 28 August 2021. Retrieved records were exported to the Covidence software [15] for deduplication [16] and screening. Both title and abstract screening and full-text review of eligibility were carried out independently by two reviewers, with disagreement resolved by discussion and involvement of a third reviewer.

Data on the characteristics of included studies were extracted by a single reviewer (DH) using a pre-designed data extraction template, which was pre-tested by two reviewers (DH and HH). An amended version of dimensions of PCT identified in a previous review [8] was used to categorise PCT ‘components’.

Counts and proportions were used to summarise the article characteristics. Thematic synthesis was employed to identify the key findings relating to barriers and facilitators. Themes were identified independently by four authors (DH, HH, ED and SWM) and refined by consensus.

## Results

**Fig. 1:**
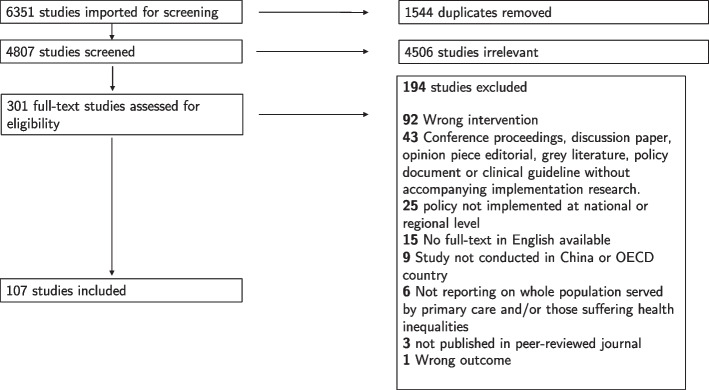
Study selection for a scoping review

A total of 6351 records were identified by the search strategy of which 1544 were duplicates. Following title and abstract screening, 301 articles underwent full-text screening with 107 articles included (Fig. 1) [6–8, 17–120].

Fifty-four per cent of the included studies employed quantitative methods alone, 28% qualitative methods alone, and 14% mixed methods, and 4% were reviews of the literature. Included studies were conducted in 14 of 39 OECD plus China, with six countries (the USA, China, Canada, the UK, Australia, and Sweden) accounting for over 80% of studies (Additional file 1: Fig. S1). Two studies examined PCT across multiple jurisdictions in Australia, Canada, and the USA [27, 28]. Characteristics of all included studies are shown in Additional file 1: Table S3 [6–8, 17–120].

**Fig. 2:**
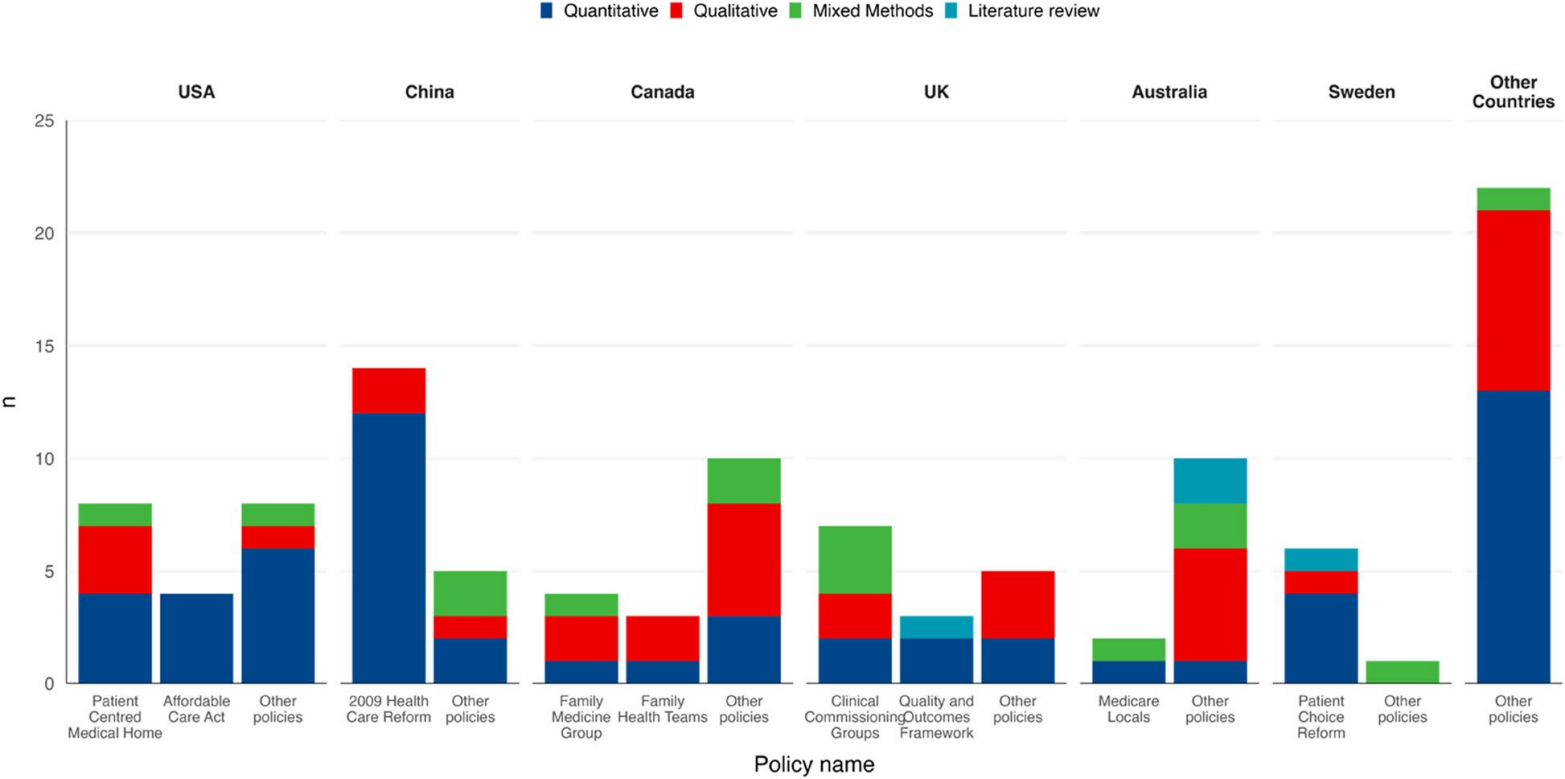
Studies evaluating particular primary care transformation policies

Figure 2 shows the specific PCT policies evaluated in included studies for the six countries contributing 80% of the included studies and the remaining nine countries aggregated. The most frequently studied policies were the 2009 Health Care Reform in China (*n* = 14), the Patient-centred medical home (PCMH) in the USA (*n* = 8), and the Clinical Commissioning Groups (CCGs) in the UK (specifically, England) (*n* = 7). Slightly over half of the studies (*n* = 59 [55%]) evaluated the policies that were unnamed or appeared only once.

The most frequently described components of PCT were expansion of the MDT (*n* = 49 [46%]), alternative payment mechanisms (*n* = 45 [42%]), and increased access to primary care (*n* = 40 [37%]) (Table 1 and Additional file 1: Fig. S2). Almost all PCT policies included multiple components. For example, 21 studies (42%) including an expansion of MDT component also included alternative payment mechanisms and/or increased primary care access. The 2009 Health Care Reform in China accounted for 22% of studies describing alternative payment mechanisms and 33% of studies describing increased primary care access. Otherwise, described components of PCT were distributed across countries and policies (Additional file 1: Figs. S2 and S3).

**Table 1:**
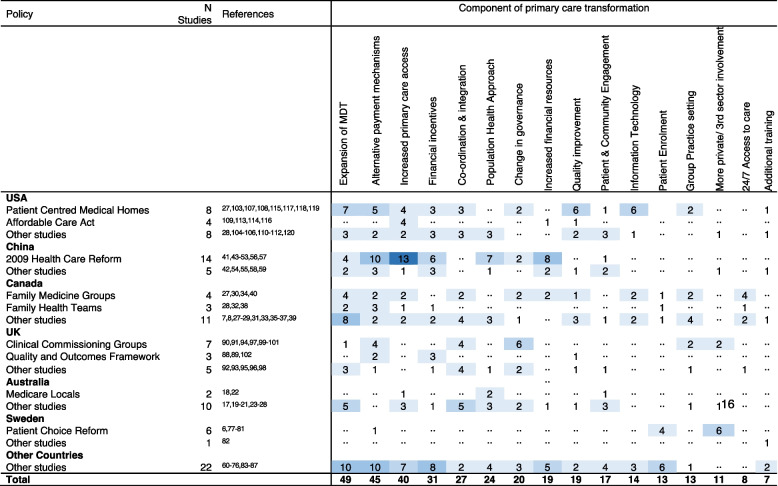
Count of included studies evaluating each component of primary care transformation, by country and policy

Of the 20 studies conducted in the USA [27, 28, 103–120], eight evaluated PCMH with PCT components including expansion of the MDT, quality improvement, and information technology (Table 1) [27, 103, 107, 108, 115, 117–119]; four evaluated the Affordable Care Act (ACA), which primarily aimed to increase primary care access [109, 113, 114, 116]; and the remaining eight evaluating other policies not replicated in other studies. The majority (14/19) of studies conducted in China evaluated the 2009 Health Care Reform and associated policies including the National Essential Medicines Policy and the New Rural Cooperative Medical Scheme [41, 43–45, 47–53, 56, 57, 111]. Components of this reform included increased primary care access, alternative payment mechanisms, and increased financial resources (Table 1). Four Canadian studies explicitly identified Family Medicine Groups (FMGs) incorporating a wide range of PCT components (Table 1) [27, 30, 34, 40]. Family Health Teams (FHTs) were mentioned in three studies [8, 32, 38] including evaluation of MDT expansion and alternative payment mechanisms. Notably, five studies conducted in Canada were review papers either incorporating a literature review or expert views [7, 8, 27, 28, 39]. Of the 15 UK papers, seven [90, 91, 94, 97, 99–101] evaluated CCGs with PCT components including change of governance and alternative payment mechanisms. Three studies [88, 89, 102] evaluated the Quality & Outcomes Framework (QOF) which included alternative payment mechanism and financial incentives. Half of papers including evaluation of Australian PCT did not name a specific policy [19–22, 25, 26]. These papers aimed to give an overview over a period of time or identify barriers and facilitators to implementation. Six of seven studies conducted in Sweden evaluated the Primary Health Care Choice Reform which involved an increased involvement of the private and/or third sector in the provision of primary care [6, 77–81].

A wide range of outcome measures were examined in the included studies (Table 2 and Additional file 1: Fig. S4). The most frequently used were views of GPs (*n* = 29 [27%]), views of managers (*n* = 29 [27%]), or views of other MDT members (*n* = 25 [23%]). These studies predominantly employed qualitative or mixed method methodologies (Additional file 1: Fig. S5). Twenty-nine studies (27%) used an outcome unique to that study or used in only one other included study. Examples include medication use [44, 48], continuity of care [92], and self-assessed health [49]. Fifteen studies (14%) measured patient views, and six (5%) measured patient satisfaction. The most frequently employed outcomes in quantitative studies were unique to that study or analysed the effect of PCT on health care use (Table 2, Additional file 1: Figs. S4 and S5).

**Table 2:**
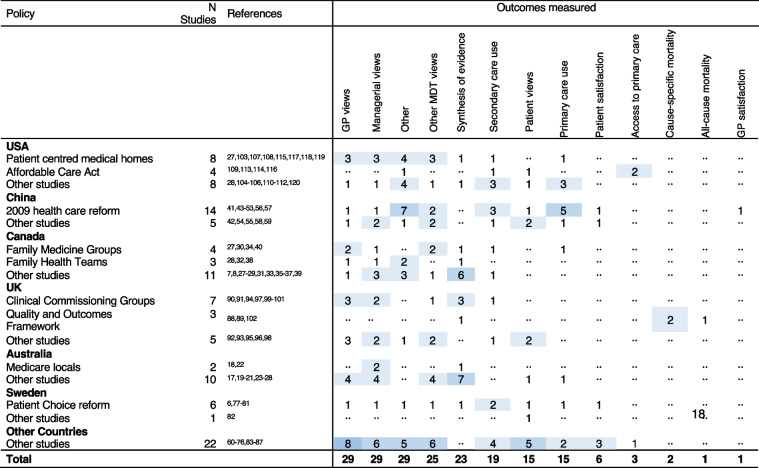
Count of outcomes evaluated in included studies, by country and policy

Twenty-seven of 29 studies [19, 21, 23, 24, 34, 37, 38, 40, 57, 58, 61–65, 71, 81, 86, 87, 90, 95–98, 101, 117–120] including GP views as an outcome also measured either managerial views (23 studies), other MDT views (23 studies), or patient views (5 studies). Three studies evaluated the aspects of the PCMH in the USA and found difficulties in recruiting MDT members [118], anticipated staff satisfaction [117], and identified important training to assist with PCMH implementation (Table 2) [119]. One study found low satisfaction among GPs following the 2009 Healthcare Reform in China [57]. Two studies including GP views in Canada reported negative effects following implementation of FMGs [34, 40]. In the UK, three studies included the views of GPs on CCGs and found concerns about outsourcing support functions due to potential loss of knowledge or funding [90, 97, 101].

Six studies measured patient satisfaction. All reported improvements in satisfaction following reforms in China [50, 54], the Netherlands [68], Turkey [83, 84], and Sweden [79]. Qualitative studies including patient views did not report patient-specific outcomes but focussed on staff performance or health care delivery findings [56, 63, 64, 86, 96]. One exception was found in Portugal where mixed results in terms of patient views towards implementation of primary health care reform were reported [76]. Other quantitative studies found positive patient perceptions of change following the introduction of the ACA in the USA [113] and negative views of a voucher scheme introduced in Hong Kong [42]. One UK study found that larger practice size may be associated with poorer continuity of care and that collaborative working among practices had no effect on patient experience [93].

Nineteen studies included measurement of primary or secondary health care use [30, 33, 43, 51, 53, 55, 66, 69, 73, 79, 80, 83, 91, 92, 105, 107, 110, 116, 120]. Only one of these (conducted in IL, USA) reported a positive benefit of PCT of reduced secondary care use [105]. One study [107] evaluating PCMH in the USA found an association with reduced hospitalisations, despite higher emergency department use. Another study evaluating the ACA found no change in preventable hospitalisations [116]. Five studies evaluating the 2009 Health Care Reform in China [41, 43, 47, 51, 53] found a desired increase in primary care use but also increased secondary care use, particularly in rural areas, reductions in out-of-pocket expenditure for outpatients although not inpatients, and high levels of inappropriate hospital use. One study evaluating FMGs in Canada found no improvement in equity of access to primary care or secondary care use [30]. In the UK, one study noted the implementation of CCGs did not result in reduced hospitalisations and, perversely, noted increased GP-referred specialist clinic visits [91].

Thirty-seven studies (35%) evaluated PCT in the context of ageing populations or health inequalities although only three specifically evaluated the former (Additional file 1: Fig. S6). Twenty reported on outcomes of PCT for those living in deprived areas [6, 21, 24, 25, 30, 31, 48, 49, 52, 63, 69, 77, 78, 83, 88, 95, 102, 110, 116, 117]. Three of these [48, 49, 52], reporting on the Chinese 2009 Health Care Reform, showed increased primary care access, particularly in low-income areas, and increased demand for services. One [49] reported an improvement in health equity; however, another [48] noted that out-of-pocket expenditure rose. Seventeen other studies conducted in Australia, Canada, Ireland, New Zealand, Sweden, Turkey, the UK, and the USA reported ambiguous results or worsening health inequalities for deprived populations [63].

The three studies evaluating PCT in the context of older people reported high levels of satisfaction with a new management pathway for older people in southwest China [54], negative outcomes (including worse continuity-of-care) following the introduction of a named GP policy in England [92], and increased access favouring younger rather than older people from more private sector involvement in primary care delivery in Sweden [77].

Ten studies evaluated PCT in light of urban/rural inequalities. Six of these were conducted in China [41, 43, 45, 55, 56, 111]: two reported positive outcomes particularly increased primary care access in rural areas [41, 43]; three reported negative outcomes, including poor GP satisfaction [45], a ‘brain-drain’ of doctors from rural to urban areas [49], and differences between provinces leading to regional disparity [111]. A more recent study in China reported mixed results [55]. Elsewhere, one study evaluating expanded primary care in Turkey [83] reported improved access, satisfaction, and service quality in rural areas. Studies in Portugal [75] and Australia [20] highlighted the difficulty in the implementation of centrally designed PCT policies in geographically dispersed populations. One UK study found a non-significant effect of QOF on mortality in urban or rural areas [88].

Thirteen studies evaluating PCT implementation in the context of ethnic minorities were conducted in Australia [20, 23–26], New Zealand [69, 70], the UK [88, 102], the USA [114, 117], Canada [36], and Sweden [82]. Positive outcomes for ethnic minorities, including increased access and quality-of-care, were reported in three studies [23, 70, 82], whilst six studies reported negative, mixed, or equivocal results [24, 25, 69, 88, 102, 114]. The remaining studies focused on service, rather than patient outcomes [20, 26, 36, 117].

Forty-one studies (38%) explicitly identified barriers or facilitators to PCT implementation, of which 59% were conducted in Canada, Australia, or the USA. The majority were qualitative or mixed method studies (Additional file 1: Fig. S7). Thematic analysis of these studies identified four main themes: (a) leadership, policy, and communication; (b) change, culture, and relationships; (c) resources and capacity; and (d) targets, outcomes, and measurement (Table 3).

**Table 3:**
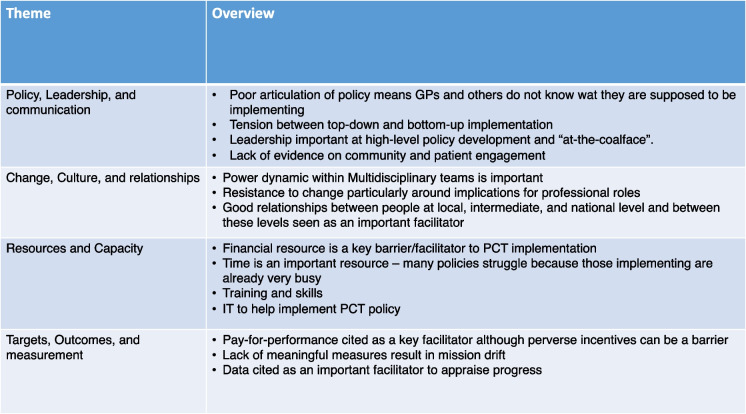
Barriers and facilitators to implementing primary care transformation policies

In the leadership, policy, and communication theme, 12 studies noted the importance of leadership in PCT implementation [27, 34, 39, 58, 63–66, 81, 101, 117, 119] which was required at higher organisational level [58, 66, 81, 101, 119] as well as at the level where services are delivered (e.g. in GP practices) [27, 34, 63]. The importance of institutional [58, 81] and personal leadership was highlighted, and the latter cited as a key facilitator ‘at-the-coalface’ [34, 39, 39, 63, 64]. Twelve studies highlighted the importance of planning and articulation of policy intentions to those delivering the initiative [18, 21, 36–38, 58, 63, 65, 72, 87, 99, 117]. This was cited as an important element when there were difficulties implementing a policy [18, 21, 36, 58, 72, 87, 99] and also in positive examples [37, 38, 63, 65, 117]. The correct balance between ‘top-down’ and ‘bottom-up’ initiatives was discussed in seven studies [7, 18, 21, 27, 37, 58, 117]. Three studies highlighted a lack of engagement where study participants perceived ‘top-down’ PCT implementation [7, 21, 37]. However, wholly ‘bottom-up’ approaches increased the risk of variable implementation across areas [18], despite the potential for greater engagement [117] and flexibility at a local level [7, 27]. Two studies found a blend of ‘top-down’ and ‘bottom-up’ approaches was likely to yield best results [18, 58].

In the second theme of change, culture, and relationships, reluctance or resistance to change among clinicians was identified as a barrier to PCT [21, 24, 37, 98, 101, 117]. Some studies reported concerns over potential reductions in primary care funding [37, 101] or change in professional roles resulting in loss of skills [98]. However, one study noted where perceived benefits to health care professionals were known in advance, change was not resisted [61]. Three studies noted the influence of power held by physicians and their representative bodies on the likelihood of PCT implementation [7, 40, 60]. Two studies [37, 117] observed any type of reform involves politics and should be planned for at the design stage of major policy change. The importance of personal relationships between and within different administrative, and/or clinical levels or in different jurisdictions, from central policy development, through local government or health authority, to direct clinical care, was highlighted as critical to the success or failure of PCT implementation in seven studies [18, 38, 39, 51, 58, 75, 81]. There was evidence of tension between PCT policies aiming to improve population health whilst primary care often prioritises chronic disease management at the individual level [21, 24, 95]. This was particularly difficult where PCT involved cross-sectoral integration or cooperation [51, 95]. Four studies observed power struggles between GPs and other clinical professionals where roles and responsibilities were changing [27, 34, 35, 61], whilst six cited positive collaboration within teams as an important facilitator to change [18, 35, 37, 38, 62, 63].

The third barrier and facilitator theme was resources and capacity. Nine studies highlighted lack of financial commitment as a fundamental barrier to PCT implementation [7, 35, 42, 51, 58, 62, 65, 72, 117] whilst two cited adequate funding as a facilitator [36, 64]. Four studies noted that time required from already very busy clinicians to adapt and implement policy goals was often overlooked [26, 62, 63, 98], whilst two noted failure to account for the additional time required in geographically dispersed areas [75]. The lack of training was identified as a barrier to implementation in four studies [24, 26, 61, 119], whereas two studies cited education and training as critical facilitators [39]. Lastly, the provision of information technology and technical assistance enabling management and measurement was cited as a key barrier/facilitator [75, 115, 117].

In the final theme of targets, outcomes, and measurement, four studies identified pay-for-performance incentives as a facilitator to support PCT implementation [38, 58, 66, 115]. However, three other studies noted these can act as a perverse incentive; for example, GPs being paid on a fee-for-service basis where wider MDT working was a policy aim [35, 40, 71]. Eight studies reported the identification and measurement of tangible outcomes as a crucial facilitator of PCT [36, 51, 63, 64, 81, 94, 99, 117]. Three studies indicated the importance of good data collection and management in order to enable this [20, 36, 75].

## Discussion

This systematic scoping review of PCT included 107 peer-reviewed studies from 15 countries and has characterised research findings on components of PCT, outcomes used to measure PCT, health inequalities and ageing populations, and barriers and facilitators to implementation. Primary care transformation is a widespread policy trend internationally. This review investigated the extent to which evidence supports the effectiveness of PCT, especially in contexts of health inequalities and ageing populations.

Over 80% of included studies were conducted in the USA, China, Canada, the UK, Australia, or Sweden. The most frequently employed components of PCT were the expansion of the MDT, alternative payment mechanisms, and increased primary care access. The most frequently measured outcomes were GP, managerial, or other MDT views, with patient perspectives and clinical or other harder outcomes not commonly examined.

Eighteen studies evaluated PCT in the context of deprived populations with mixed results. Benefits were largely found in countries with less-developed primary care systems where access was expanded. Nine studies reported no change or widening health inequalities following PCT implementation. Two of three studies evaluating PCT in the context of ageing populations found negative impacts including worsening continuity of care and access to primary care.

Thematic analysis of 41 studies citing barriers or facilitators to implementation of PCT identified four themes: (a) leadership, policy, and communication; (b) change, culture, and relationships; (c) resources and capacity; and (d) targets, outcomes, and measurement. Clear articulation of intended outcomes to those working ‘at-the-coalface’ is more likely to result in positive implementation.

Jimenez et al. [9] conducted a systematic review of 37 studies with only one database (MEDLINE) but also including a search of grey literature. They focused on multi-component interventions, required a comparator, and defined a set of outcomes for included studies. Unlike our review, they did not examine the inequalities or barriers/facilitators to PCT implementation, although they also found some increases in primary care access and mixed results on the effect of PCT on secondary care use. One other scoping review [10], with an unreported number of included studies, presented findings regarding the importance of GP engagement with policy changes and the importance of power dynamics within MDTs. Miller et al. [10] also found lack of community engagement with PCT design was due to health care professionals lacking time and/or capacity. Our review adds strength to both of these findings but additionally finds that good communication of PCT aims with measurable outcomes can negate issues of engagement.

Our review has three main limitations. Firstly, components of PCT were counted as reported in included papers. Some reforms may have other components not described and were not included in our counts. Secondly, we excluded grey literature and non-peer-reviewed research articles from our search strategy, although this may have minimal impact since Jimenez et al. [9] did not identify any additional studies from searching the grey literature. Finally, our review included only English-language papers resulting in 15, predominantly Chinese, papers being excluded from the full-text review.

There are three key implications for practice or policy from our review. The expansion of MDT to include a range of non-medical health care professionals in primary care delivery was a fundamental component of reform across many countries, regardless of the primary care system. This is consistent with the WHO guidelines [1, 5]. However, we found no evidence to describe the effectiveness of this PCT component on outcomes. MDT working can be hindered by a lack of training, resistance to change, and poor professional relationships. These factors all have an economic dimension to them; for instance, primary care in more straightened circumstances can entail clinicians who are too busy to undertake additional training even if recommended and available, who can be expected to disengage with policies that entails time to rework practices to align with them, and who might further lack the time to invest in building, maintaining, and strengthening inter-professional relationships. Policymakers should ensure PCT planning includes sufficient resources to enable implementation, including training of MDT members and development of quality inter-professional relationships, and that ensure clinicians are active partners in the process of care.

Secondly, an often-stated aim of PCT is to increase primary care utilisation and efficiency whilst simultaneously reducing secondary and unscheduled care utilisation [2]. Our review found no evidence that any PCT policy has substantially achieved this twin aim. There were notable increases in primary care use in countries with historically low levels of utilisation, such as China or Turkey, following the implementation of policies designed to achieve this aim. For countries with more developed access to primary care, this lack of evidence means questions remain as to whether and how PCT will reduce secondary care utilisation, particularly in the face of ageing populations and rising needs.

Thirdly, the review highlighted the importance of well-articulated policies with committed leadership at all levels of implementation and clear targets, outcomes, and means of measurement. This involves excellent working relationships between actors within and across all levels of implementation. Adequate financial resourcing of PCT and an acknowledgement of the time that health care professionals will require to implement change are also important facilitators. Those seeking to design PCT policy need to find a balance between central control to maintain consistency across jurisdictions, whilst also enabling flexibility to enable adaptation at a local level. This may be particularly important for geographically dispersed populations or other populations with distinctive needs.

Outcomes in the review were dominated by health care professional views or health care use. Future studies should consider PCT for patient-related outcomes, such as continuity-of-care, or from a quality-of-care perspective. Future research should also consider the dual impact of PCT on older populations and on health inequalities. We found little evidence evaluating the former and mixed results for the latter.

## Conclusions

Successful implementation of PCT relies on good relationships and clear understanding of roles between GPs and other health care professionals. More generally, leadership at all levels, financial commitment, and policy design, including both ‘top-down’ and ‘bottom-up’ approaches, appear to facilitate successful implementation. However, there is a lack of evidence on the effects of PCT, both in general and specifically for older people, and on health inequalities, with few studies evaluating the impact using patient-related or quality of care outcomes.

### Supplementary Information

**Additional file 1:** **Table S1.** PRISMA-ScR Checklist. **Table S2.** MEDLINE Search terms. **Table S3.** Characteristics of included studies. **Fig. S1.** Included studies by country and methodology. **Fig. S2.** Count of types of primary care transformation in included studies, by policy. **Fig. S3:** Count of types of primary care transformation in included studies, by policy (alt). **Fig. S4.** Count of outcome measures in included studies, by policy. **Fig. S5.** Outcome measures in included studies, by methodology. **Fig. S6.** Types of health inequalities measured (*n*=37). **Fig. S7.** Studies citing barriers and facilitators to PCT (*n*=41), by methodology and country. **Fig. S8.** Included studies by year 

## Data Availability

All data generated or analysed during this study are included in this published article [and its supplementary information files].

## Abbreviations

ACA: Affordable Care Act
CCGs: Clinical commissioning groups
CINAHL: Cumulative Index for Nursing and Allied Healthcare Literature
FHTs: Family Health Teams
FMGs: Family Medicine Groups
GP: General practitioner
MDT: Multidisciplinary team
OECD: Organisation for Economic Cooperation and Development
OSF: Open Science Framework
PCMH: Patient-centred medical home
PCT: Primary care transformation
PICO: Population, Intervention, Comparator, and Outcome
PRISMA: Preferred Reporting Items for Systematic Reviews and Meta-Analyses
QOF: Quality and Outcomes Framework
WHO: World Health Organization
