# Supplementary material for: Understanding primary care transformation and implications for ageing populations and health inequalities: a systematic scoping review of new models of primary health care in OECD countries and China
Source: BMC Med. 2023 Aug 24;21:319. doi: 10.1186/s12916-023-03033-z (PMC10463288; doi:10.1186/s12916-023-03033-z)

Understanding primary care transformation and implications for ageing populations and health inequalities: a systematic scoping review of new models of primary health care in OECD countries and China

Supplementary material

Table of Contents

Table of Contents	Page(s)
**Table S1: PRISMA-ScR Checklist**	**2**
**Table S2: MEDLINE Search terms**	**4**
**Table S3: Characteristics of included studies**	**6-93**
**Supplementary Figures 1-8**	**94-101**

Table S1: Preferred Reporting Items for Systematic reviews and Meta-Analyses extension for Scoping Reviews (PRISMA-ScR) Checklist

SECTION	ITEM	PRISMA-ScR CHECKLIST ITEM	REPORTED ON PAGE #
**TITLE**			
Title	1	Identify the report as a scoping review.	1
**ABSTRACT**			
Structured summary	2	Provide a structured summary that includes (as applicable): background, objectives, eligibility criteria, sources of evidence, charting methods, results, and conclusions that relate to the review questions and objectives.	2
**INTRODUCTION**			
Rationale	3	Describe the rationale for the review in the context of what is already known. Explain why the review questions/objectives lend themselves to a scoping review approach.	5
Objectives	4	Provide an explicit statement of the questions and objectives being addressed with reference to their key elements (e.g., population or participants, concepts, and context) or other relevant key elements used to conceptualize the review questions and/or objectives.	5
**METHODS**			
Protocol and registration	5	Indicate whether a review protocol exists; state if and where it can be accessed (e.g., a Web address); and if available, provide registration information, including the registration number.	5
Eligibility criteria	6	Specify characteristics of the sources of evidence used as eligibility criteria (e.g., years considered, language, and publication status), and provide a rationale.	5 & 6
Information sources*	7	Describe all information sources in the search (e.g., databases with dates of coverage and contact with authors to identify additional sources), as well as the date the most recent search was executed.	6
Search	8	Present the full electronic search strategy for at least 1 database, including any limits used, such that it could be repeated.	Supplementary material
Selection of sources of evidence†	9	State the process for selecting sources of evidence (i.e., screening and eligibility) included in the scoping review.	6
Data charting process‡	10	Describe the methods of charting data from the included sources of evidence (e.g., calibrated forms or forms that have been tested by the team before their use, and whether data charting was done independently or in duplicate) and any processes for obtaining and confirming data from investigators.	6
Data items	11	List and define all variables for which data were sought and any assumptions and simplifications made.	6
Critical appraisal of individual sources of evidence§	12	If done, provide a rationale for conducting a critical appraisal of included sources of evidence; describe the methods used and how this information was used in any data synthesis (if appropriate).	NA
Synthesis of results	13	Describe the methods of handling and summarizing the data that were charted.	6

SECTION	ITEM	PRISMA-ScR CHECKLIST ITEM	REPORTED ON PAGE #
**RESULTS**			
Selection of sources of evidence	14	Give numbers of sources of evidence screened, assessed for eligibility, and included in the review, with reasons for exclusions at each stage, ideally using a flow diagram.	6 & Figure 1
Characteristics of sources of evidence	15	For each source of evidence, present characteristics for which data were charted and provide the citations.	Supplementary Table 3
Critical appraisal within sources of evidence	16	If done, present data on critical appraisal of included sources of evidence (see item 12).	NA
Results of individual sources of evidence	17	For each included source of evidence, present the relevant data that were charted that relate to the review questions and objectives.	Supplementary Table 3
Synthesis of results	18	Summarize and/or present the charting results as they relate to the review questions and objectives.	7-11
**DISCUSSION**			
Summary of evidence	19	Summarize the main results (including an overview of concepts, themes, and types of evidence available), link to the review questions and objectives, and consider the relevance to key groups.	11
Limitations	20	Discuss the limitations of the scoping review process.	12
Conclusions	21	Provide a general interpretation of the results with respect to the review questions and objectives, as well as potential implications and/or next steps.	13
**FUNDING**			
Funding	22	Describe sources of funding for the included sources of evidence, as well as sources of funding for the scoping review. Describe the role of the funders of the scoping review.	3

JBI = Joanna Briggs Institute; PRISMA-ScR = Preferred Reporting Items for Systematic reviews and Meta-Analyses extension for Scoping Reviews.

* Where *sources of evidence* (see second footnote) are compiled from, such as bibliographic databases, social media platforms, and Web sites.

† A more inclusive/heterogeneous term used to account for the different types of evidence or data sources (e.g., quantitative and/or qualitative research, expert opinion, and policy documents) that may be eligible in a scoping review as opposed to only studies. This is not to be confused with *information sources* (see first footnote).

‡ The frameworks by Arksey and O'Malley (6) and Levac and colleagues (7) and the JBI guidance (4, 5) refer to the process of data extraction in a scoping review as data charting.

§ The process of systematically examining research evidence to assess its validity, results, and relevance before using it to inform a decision. This term is used for items 12 and 19 instead of "risk of bias" (which is more applicable to systematic reviews of interventions) to include and acknowledge the various sources of evidence that may be used in a scoping review (e.g., quantitative and/or qualitative research, expert opinion, and policy document).

From: Tricco AC, Lillie E, Zarin W, O'Brien KK, Colquhoun H, Levac D, et al. PRISMA Extension for Scoping Reviews (PRISMAScR): Checklist and Explanation. Ann Intern Med. 2018;169:467–473. doi: 10.7326/M18-0850.

Table S2: Medline Search strategy

#	Search Term	Hits
1	Primary Health Care/	166817
2	physicians, family/	112893
3	physicians, primary care/	105844
4	Patient Care Team/ and (primary or community).ti,ab.	41615
5	((multidisciplinary team* or multi-disciplinary team* or interdisciplinary team* or inter-disciplinary team*) and (primary or community)).ti,ab.	15073
6	((primary care or primary heathcare or primary health care or family health or family medicine) adj7 (reform or transform* or programme or program or strateg*)).ti,ab.	23908
7	Health Care Reform/ or (reform or transform*).ti,ab.	675365
8	Health Policy/	257311
9	policy/	109782
10	(national or nationwide).ab,ti.	1509341
11	(policy* or policies).ab,ti.	678665
12	(country or countries).ab,ti.	1209469
13	1 or 2 or 3 or 4 or 5	326369
14	7 and 13	14147
15	6 or 14	36039
16	8 or 9 or 10 or 11 or 12	3176727
17	15 and 16	16256
18	(Clinical Trial Protocol or Practice Guideline).pt or Feasibility Studies/	244671
19	17 not 18	16119
20	(Australia or Austria or Belgium or Canada or Chile or Colombia	9699448

	or Costa Rica or Czech Republic or Denmark or Estonia or Finland or France or Germany or Greece or Hungary or Iceland or Ireland or Israel or Italy Japan or South Korea or Latvia or Lithuania or Luxembourg or Mexico or Netherlands or New Zealand or Norway or Poland or Portugal or Slovakia or Slovenia or Spain or Sweden or Switzerland or Turkey or United Kingdom or United States or China or England or Scotland or Northern Ireland or Wales).mp	
21	19 and 20	7917
22	limit 21 to year=”2010 – Current”	4095

Table S3: **Characteristics of Included studies**

Study	Country/ Policy/ Methodology	Primary Aim	Type of PC Transformation	Main outcome measurements	Description of Population	Findings
Taylor et al (2013)[17]	Australia<br><br>May 2010 reform of Medicare Benefit Schedule (MBS)<br><br>Quantitative	To determine what types of supply-side change underpinned the recent decline in longer (Level C and D) consultation provision and to evaluate the impact of the May 2010 reforms in realigning Medicare with long-term health policy objectives.	Financial incentives	Primary care utilisation	Medicare data for all divisions of General Practice from the first quarter of 2006 until second quarter of 2011.	a) The overwhelming majority of GPs continued to provide level C consultations during the study period but did so less often<br>b) The proportion of GPs providing level D consultations decreased and they were also provided less often<br>c) Many GPs stopped providing level D consultations altogether<br>d) The reforms did not have the anticipated benefit of increasing longer level C & D consultations.

Study	Country/ Policy/ Methodology	Primary Aim	Type of PC Transformation	Main outcome measurements	Description of Population	Findings
Robinson et al (2015)[18]	Australia<br><br>Medicare Locals<br><br>Quantitative	To evaluate the development and implementation of Medicare Locals as new primary care organisations and consider the implications of these findings for the wider challenge of strengthening primary healthcare in Australia and internationally	a) Population health approach<br>b) Community engagement<br>c) Increased primary care access	Managerial views	43 Medicare Local chief executive officers	a) There was a difference in form and function of Medicare Locals suggesting the balance between local needs and retaining consistency more broadly. Some respondents suggested this was partly due to lack of national policy direction<br>b) In early stages of reform, this inconsistency led to confusion among stakeholders around the role of MLs.<br>c) Likely that a neither a top-down nor bottom-up approach is desirable but will require a combined approach

Study	Country/ Policy/ Methodology	Primary Aim	Type of PC Transformation	Main outcome measurements	Description of Population	Findings
Jolley et al (2014)[19]	Australia<br><br>No specified policy<br><br>Qualitative	To examine recent Australian health reform policies and consider how the primary health care workforce experienced subsequent change and perceives its impact on health promotion practice.	a) Population health approach<br>b) Group practice setting	a) GP views<br>b) Other MDT views<br>c) Managerial views	39 managers and health practitioners from four South Australian primary health care services	a) Numerous reforms from 2003 to 2010 stipulate health promotion as an aim of reform<br>b) Those interviewed in the study reported there have been fewer opportunities to practice health promotion over the same period<br>c) This is due to reforms also directly targeting individual behaviour change

Study	Country/ Policy/ Methodology	Primary Aim	Type of PC Transformation	Main outcome measurements	Description of Population	Findings
Lyle et al (2017)[20]	Australia<br><br>No specified policy<br><br>Qualitative	To report on the key findings from seven Centre for Research Excellence service evaluations to better understand what made these primary health care models work where they worked, and why.	a) Community engagement<br>b) Service coordination and integration<br>c) Change in governance<br>d) Increased primary care access	Thematic synthesis of literature	Narrative synthesis of 15 articles reporting on seven service evaluations of different primary health care models published between 2012 and 2015.	a) Community ownership and leadership were reported as key drivers for significant changes to primary health care service provision in two community-based models<br>b) Establishing new rural and remote services required either significant redesign of existing services and/or the addition of new service elements to address service gaps<br>c) Routine collection of data was employed to ensure ongoing provision, high-quality care and to inform service adaption

Study	Country/ Policy/ Methodology	Primary Aim	Type of PC Transformation	Main outcome measurements	Description of Population	Findings
Javanparast et al (2017)[21]	Australia<br><br>No specified policy<br><br>Qualitative	To present a case-study of primary health care restructuring and how these changes have been managed from the viewpoint of practitioners and middle managers	a) Population health approach<br>b) Multi-disciplinary teams<br>c) Service coordination and integration<br>d) Increased primary care access	a) GP views<br>b) Other MDT views<br>c) Managerial views	57 practitioners and 6 regional and central health executives from 7 study sites in South Australia.	a) Various reforms have led to a shift of focus from health promotion and community-based activities to chronic disease management and clinical and individually-based treatment<br>b) Policy changes were top-down radical reforms with minimal communication to practitioners and service managers<br>c) Services with a community-controlled model of governance had more autonomy to employ change

Study	Country/ Policy/ Methodology	Primary Aim	Type of PC Transformation	Main outcome measurements	Description of Population	Findings
Javanparast et al (2018)[22]	Australia<br><br>Medicare Locals<br><br>Mixed Methods	To describe how institutional forces, ideas and actors shaped population health planning via Medicare locals in Australian primary health care	Population health approach	a) Managerial views<br>b) Thematic synthesis of literature	61 planning documents for Medicare locals. 50 Medicare local senior staff, four Federal Department of Health staff	a) Despite policy emphasis on population health, health promotion and social determinants of health activities were undertaken on an ad hoc basis<br>b) Regulatory conditions imposed by the federal government including funding priorities and time schedules were the predominant forces constraining population health planning

Study	Country/ Policy/ Methodology	Primary Aim	Type of PC Transformation	Main outcome measurements	Description of Population	Findings
Javanparast et al (2018)[23]	Australia<br><br>No specified policy<br><br>Mixed Methods	To examine the scope and potential value for community health workers to improve health equity in Australia.	a) Multi-disciplinary teams<br>b) Community engagement	a) GP views<br>b) Other MDT views<br>c) Managerial views<br>d) Thematic synthesis of literature	47 documents relevant to community health workers in Australia and interviews with two policy-makers, six program managers and three practitioners.	a) Community health workers foster trust, community connection, advocacy and bridging communities to formal health systems which are core to improving healthcare access and equity - especially for disadvantaged groups<br>b) Community health workers can perform a broad range of health-related functions<br>c) There is no single definition of Community Health Workers in Australia and no clarity on role or scope of practice

Study	Country/ Policy/ Methodology	Primary Aim	Type of PC Transformation	Main outcome measurements	Description of Population	Findings
Baum & Freeman (2021)[24]	Australia<br><br>Comprehensive Primary Health Care (CPHC); Medicare Locals and Primary Health Networks (PHNs)<br><br>Mixed Methods	To examine the factors that account for the absence of strong community health systems in high income countries, using Australia as an example	a) Population health approach<br>b) Multi-disciplinary teams<br>c) Community engagement<br>d) Service coordination and integration<br>e) Change in governance	a) Patient views<br>b) GP views<br>c) Other MDT views<br>d) Managerial views<br>3) Thematic synthesis of literature	a) Seven community health services.<br>b) Staff and board members from PHNs and Medicare Locals (N not reported)<br>c) Federal Department of Health staff (N not reported)<br>d) Aboriginal and Torres Strait Islander, migrant, and mental health organization representatives (N not reported).	a) CPHC services were effective at prioritising populations with most need e.g. Aboriginal and Torres Strait Islanders and those on low incomes. Move to PHNs via Medicare Locals has resulted in loss of this focus<br>b) Medicare locals and PHNs are dominated by a medical model of health which reduces possibility of Community health services
Fisher et al (2017)[25]	Australia<br><br>No specified policy<br><br>Literature review	To identify key recent changes in national primary health care (PHC) policy and assess implications for equity of access to PHC.	a) Service coordination and integration<br>b) Increased primary care access<br>c) Increased private and/or third sector involvement	Thematic synthesis of literature	909 academic papers and 90 grey literature items relating to Australian PHC policy reviewed. Unclear how many included in narrative synthesis.	a) Shift to Medicare Benefits Schedule rebates poses risk to equity of access to PHC as may result greater uptake by more affluent populations<br>b) Increased involvement of private healthcare in PHC poses significant potential risk to equity of access to care

Study	Country/ Policy/ Methodology	Primary Aim	Type of PC Transformation	Main outcome measurements	Description of Population	Findings
Gardner et al (2018)[26]	Australia<br><br>No specified policy<br><br>Literature review	To explore uptake and implementation in indigenous primary health care, including barriers and enablers, to embedding continuous quality improvement (CQI) in routine practice.	Continuous performance measurement and quality improvement	Thematic synthesis of evidence	60 academic and grey literature papers	a) Barriers to implementing CQI relate primarily to professional and organisational change processes and operate at multiple levels (individual, team, service, and health system)<br><br>b) Barriers to improved carer relate more to directly to knowledge of best practice and team processes that facilitate appropriate care such as multidisciplinary teamwork for complex conditions, adequate staffing, financial incentives etc.

Study	Country/ Policy/ Methodology	Primary Aim	Type of PC Transformation	Main outcome measurements	Description of Population	Findings
Harris et al (2016)[27]	Australia; Canada; USA<br><br>Enhanced primary care; Patient-centred medical home; primary care networks: blended payments; Family health teams, Family medicine groups.<br><br>Qualitative	To assess the impact of reform policies and interventions that have aimed to create or enhance teamwork on professional communication relationships, roles, and work satisfaction in primary healthcare practices by synthesising evidence from comparable studies in three countries.	a) Group practice setting<br>b) Multi-disciplinary teams<br>c) Service coordination and integration	Synthesis of evidence	12 researchers that were major contributors to primary care practice-based qualitative and quantitative studies from Australia, Canada and the United States	a) The impact on communication and relationships between different professional groups, the roles of nursing and allied health services, and the work satisfaction of primary health care providers varied more within than between jurisdictions<br>b) The variation in these outcomes was associated with local factors such as the size, power dynamics, leadership, and physical environment of the practice<br>c) Reforms sometimes resulted in conflict between medical and non-medical professional groups<br>d) Not easy to predict impacts of reform. Some characteristics can be both enabling and disabling e.g. larger practices may have less capacity for adoption but more capacity to support interprofessional practice.

Study	Country/ Policy/ Methodology	Primary Aim	Type of PC Transformation	Main outcome measurements	Description of Population	Findings
Russell et al (2018)[28]	Australia; Canada; USA<br><br>No specified policy<br><br>Qualitative	To synthesis findings across five jurisdictions in three countries to identify common contextual factors influencing the successful implementation of teamwork with primary care practices.	Multi-disciplinary teams	Synthesis of evidence	Investigators on 12 published studies relevant to team working in primary care.	Seven common levers across the three countries were found to influence a jurisdiction's ability to implement interprofessional teams in primary care 1. Funding that extended beyond fee-for-service.<br>2. Care delivery that did not require direct physician involvement.<br>3. Governance that was inclusive of non-physician disciplines.<br>4. Positive attitude to team-based care from health professional organisations.<br>5. External accountability required of primary care practices<br>6. Good links with local community and medical peers<br>7. Programs involving outreach facilitations, leadership training and financial support for team activities.

Study	Country/ Policy/ Methodology	Primary Aim	Type of PC Transformation	Main outcome measurements	Description of Population	Findings
Lavergne et al (2014)[29]	Canada<br><br>Full-service family practice<br><br>Quantitative	Using population-based and provider-specific administrative data, to operationalise the dimensions of full-service family practice as defined by the British Columbia Medical Associations and describe provision of healthcare services reflecting these dimensions before, during and after reform efforts.	a) Group practice setting<br>b) Financial incentives<br>c) Additional training for healthcare practitioners	Average annual change.	2,732 - 3,429 physicians in British Columbia in the years 1991/92 - 2009/10	a) Reform efforts are not achieving their intended aims. Access, continuity and coordination of care fell over the study period<br>b) Comprehensiveness of care indicators for obstetrics and geriatric care also fell<br>c) Other comprehensiveness of care indicators did not change significantly<br>d) Declining trends were consistent across physician characteristics.

Study	Country/ Policy/ Methodology	Primary Aim	Type of PC Transformation	Main outcome measurements	Description of Population	Findings
Ouimet et al (2015)[30]	Canada<br><br>Family Medicine Groups (FMGs); Network clinical<br><br>Quantitative	To asses the impact of the primary health care reform on equity by examining the association between socioeconomic status (SES) and utilisation of healthcare services between 2003 and 2010; and to determine how the organisational model of primary health care facilities impact utilisation of services according to SES.	a) Multi-disciplinary teams<br>b) Patient enrolment choice<br>c) 24/7 access to care<br>d) Service coordination and integration	a) Primary care utilisation<br>b) Non-primary care healthcare utilisation	9206 and 9180 respondents to population surveys in 2005 and 2010 respectively.	a) Compared to lowest SES, highest SES was associated with less emergency room visits, higher likelihood of at least one visit to a primary health care facility, lower likelihood or frequent visits to primary health care and higher affiliation to a family doctor<br>b) Differences between high and low SES remained stable between 2005 and 2010 suggesting the inequities in access to primary health care did not improve following the implementation of new models of primary care

Study	Country/ Policy/ Methodology	Primary Aim	Type of PC Transformation	Main outcome measurements	Description of Population	Findings
Lofters et al (2018)[31]	Canada<br><br>Enhanced fee-for-service model<br><br>Quantitative	To evaluate whether Ontario's enhanced fee-for-service model was associated with a change in the gaps in cancer screening for people living with low income and people who are foreign-born	a) Population health approach<br>b) Alternative payment mechanisms<br>c) Financial incentives<br>d) Continuous performance measurement and quality improvement<br>e) Increased primary care access	Cancer screening uptake	Patients of 7336 family physicians who transitioned from traditional fee-for-service to a Patient Enrolment model from 2002 to 2013	a) Cancer screening was consistently lower among immigrants and among people in the lowest income quintile throughout the study period<br>b) Transition to enhanced fee-for-service was generally associated with increasing screening uptake for all<br>c) However, there was a widening of the gap in cancer screening between immigrants and long-term residents and between people living in the lowest v highest income quintile

Study	Country/ Policy/ Methodology	Primary Aim	Type of PC Transformation	Main outcome measurements	Description of Population	Findings
Batista et al (2019)[32]	Canada<br><br>Family Health Networks (FHNs); Family Health Organisations (FHOs); Family Health Groups (FHGs)<br><br>Quantitative	To examine the levels of enrolment in traditional and reformed primary health care practices by immigration status in Ontario and identify main predictors for enrolment.	a) Alternative payment mechanisms<br>b) Financial incentives<br>c) Patient enrolment choice<br>d) 24/7 access to care<br>e) Increased primary care access	Level of patient enrolment in primary care practices	Closed cohort of 9,231,840 residents in Ontario 1985 to 2002.	a0 Overall enrolment in primary care practices increased gradually after 2004 to 2012 when 67% of the cohort were enrolled<br>b) Immigrants' enrolment was consistently lower than long-term residents over the study period<br>c) By 2012, enrolment of immigrants in capitation-based models was significantly lower, particularly in FHTs which were considered the most comprehensive care model<br>d) There is, therefore, implications for equitable primary care access for immigrant populations.

Study	Country/ Policy/ Methodology	Primary Aim	Type of PC Transformation	Main outcome measurements	Description of Population	Findings
Haj-Ali et al (2020)[33]	Canada<br><br>No specified policy<br><br>Quantitative	To investigate the relationship between receiving care from interprofessional versus non-interprofessional primary care teams and ambulatory care sensitive conditions (ACSC) hospitalisations and hospital readmissions.	Inter professional teeams	Non-primary care healthcare utilisation	2,920,990 multimorbid patients in Ontario.	a) There was no difference in change over time in ACSC admissions between interprofessional and non-interprofessional teams<br><br>b) There was no statistically significant changes in all-cause hospital readmissions between interprofessional and non-interprofessional teams

Study	Country/ Policy/ Methodology	Primary Aim	Type of PC Transformation	Main outcome measurements	Description of Population	Findings
Rodríguez & Pozzebon (2010)[34]	Canada<br><br>Family Medicine Groups (FMGs)<br><br>Qualitative	To examine the first years of implementation of the family medicine group program, with a focus on the emergence of the organisational identity of one of the pilot groups located in the urban area of Montreal	a) Multi-disciplinary teams<br>b) Alternative payment mechanisms<br>c) 24/7 access to care<br>d) Service coordination and integration<br>e) Continuous performance measurement and quality improvement<br>f) Increased primary care access	a) GP views<br>b) Other MDT views	11 clinicians and administrative staff from one FMG in Montreal, Quebec	a) Very little changed in terms of practice in the early stages of the new policy being implemented<br>b) Physicians were very busy and had little time or interest in adapting to new models. This led to power struggles between physicians and nurses<br>c) A new leader made a big impact in changing practice and enhancing interprofessional working

Study	Country/ Policy/ Methodology	Primary Aim	Type of PC Transformation	Main outcome measurements	Description of Population	Findings
Misfeldt et al (2017)[35]	Canada<br><br>No specified policy<br><br>Qualitative	To identify and review diverse evidence on the people and events shaping team-based primary healthcare alongside formal policies produced and implemented in a particular time period.	a) Group practice setting<br>b) Multi-disciplinary teams<br>c) Service coordination and integration	a) Managerial views<br>b) Thematic synthesis of literature	30 interviews with stakeholders across three Canadian provinces.	a) Strong working relationships and Physician buy-in were essential to implementing team-based care<br>b) In British Columbia, non-physician health care workers (e.g. nurses) did not have same access to policymakers as physicians and so discussion about other models of primary care delivery may be limited<br>c) Lack of resources and funding incentives mades enacting policy difficult. Fee-for-service funding models do not promote team-based care

Study	Country/ Policy/ Methodology	Primary Aim	Type of PC Transformation	Main outcome measurements	Description of Population	Findings
Suter et al (2017)[36]	Canada<br><br>No specified policy<br><br>Qualitative	To analyse and compare primary health care policies in British Columbia, Alberta and Saskatchewan to understand how they inform the design and implementation of team based primary health care service delivery.	Multi-disciplinary teams	Synthesis of evidence	3 case studies, 30 key informant interviews, Group discussion with 15 representatives from provincial ministries of health, regional health authorities, regulatory bodies and professional associations.	a) The concept of team based care varied widely across provinces<br>b) Policy gaps related to team configuration, leadership, scope of practice, role clarity and financing of team-based care<br>c) Few policies explicitly contain methods of monitoring and evaluation of team-based care<br>d) Four recommendations made; ensure alignment of goals and policies at different system levels, investement of resources for system change, compensation models for all members of the team, and accountability through collaborative practice metrics

Study	Country/ Policy/ Methodology	Primary Aim	Type of PC Transformation	Main outcome measurements	Description of Population	Findings
Hanlon et al (2019)[37]	Canada<br><br>Northern Health primary health care reform (2011)<br><br>Qualitative	To critically examine efforts to achieve primary health care reform using a consultative and relationship-building approach.	a) Group practice setting<br>b) Multi-disciplinary teams<br>c) Service coordination and integration	a) GP views<br>b) Other MDT views<br>c) Managerial views	237 key informants: Northern Health employees (managers, frontline healthcare providers) and community, non-Northern Health employee informants (GPs, community organisation reps) 2012-2015.	a) Reform perceived as "top-down" by "frontline" workers who felt they had little input to the design of change. These workers had element of emotion and ownership to deal with heavy workload and therefore solutions from "above" met with resistance<br>b) Tension between "data-driven" approach of regional policymakers and "grassroots" perspective of local workers. Policymakers saw themselves as system builders in the "big-picture" but this was often perceived as being abstract by local workers<br>c) Regional policymakers felt they were trying to strike a balance between allowing local workers to take ownership of reform without appearing to be directionless<br>d) National Health policy change was similar to a scalar network where local actors were encouraged to feel as though they were part of a relational network whilst power and authority remained unequal.

Study	Country/ Policy/ Methodology	Primary Aim	Type of PC Transformation	Main outcome measurements	Description of Population	Findings
Ashcroft et al (2019)[38]	Canada<br><br>Family Health Teams (FHTs)<br><br>Qualitative	To determine the modes of communication that were used to relay policy expectations underpinning a newly emerging interprofessional model of primary health care.	a) Multi-disciplinary teams<br>b) Alternative payment mechanisms	a) GP views<br>b) Managerial views<br>c) Key policy informant views	Seven Key policy informants and 29 FHT leaders from 5 provincial regions of Ontario	a) Varying degrees of satisfaction with the methods of communication used to convey policy change;Some participants cited good communication methods used to convey policy intention (e.g. direct one-to-one support from policy makers) as a key facilitator to the success of implementing FHTs<br>b) Mentorship and learning from peers at other FHTs also cited as a useful method for implementing policy<br>c) Financial incentives were seen as a good way to indicate policy maker intentions but can result in shifting attention away from team priorities.

Study	Country/ Policy/ Methodology	Primary Aim	Type of PC Transformation	Main outcome measurements	Description of Population	Findings
Leslie et al (2020)[39]	Canada<br><br>Primary Care Networks (PCNs)<br><br>Qualitative	To provide an implementation history of the PCNs, giving a detailed account of how people, time, and culture have interacted to implement bottom-up, incremental change in a predominantly Fee-For-Service (FFS) environment	a) Population health approach<br>b) Multi-disciplinary teams<br>c) 24/7 access to care<br>d) Service coordination and integration<br>e) Increased primary care access	a) Managerial views<br>b) Synthesis of evidence	20 relevant policy documents and 11 key stakeholders in Alberta	a) PCNs have changed nature over time from an era where local solutions were found to local problems to an era where there are central demands for standardised measures, governance, and co-planning with other elements of the health system<br>b) Both eras have seen PCNs as systems that support family physician authority and autonomy<br>c) PCNs survival and success has been due to a core group of people who developed Quality Improvement over time

Study	Country/ Policy/ Methodology	Primary Aim	Type of PC Transformation	Main outcome measurements	Description of Population	Findings
Gilbert et al (2013)[40]	Canada<br><br>Family Medical Groups (FMG)<br><br>Mixed Methods	To elucidate the change dynamics and the involvement of professionals in a primary healthcare reform initiative carried out in the Canadian province of Quebec	a0 Group practice setting<br>b) Multi-disciplinary teams<br>c) 24/7 access to care<br>d) Information technology<br>e) Change in governance<br>f) Increased financial resources in the system	a) GP views<br>b) Other MDT views<br>c) Managerial views	104 health professionals, and local and regional managers in 5 FMGs in Quebec in 2003 and 2005	a) Large-scale change is formed of multiple, differing changes at lower organisational levels<br>b) Financial incentives and fee-for-service remuneration for Physicians is a sub-optimal model in which to foster integrated and team-based care<br>c) FMGs was a voluntary policy and therefore change was limited. The medical profession has considerable power over whether to enact a policy or not

Study	Country/ Policy/ Methodology	Primary Aim	Type of PC Transformation	Main outcome measurements	Description of Population	Findings
Levesque et al (2015)[7]	Canada<br><br>No specified policy<br><br>Mixed Methods	To identify the factors that have facilitated or hindered implementation of reforms in Canadian provinces between 2000 and 2010.	a) Multi-disciplinary teams<br>b) Information technology<br>c) Continuous performance measurement and quality improvement	a) Thematic synthesis of literature<br>b) Researcher and policy-maker views	40 researchers and 20 decision-makers from five Canadian provinces	a) Primary care reform has varied in scope and implementation strategy across the 5 included provinces<br>b) The main barriers to reform were lack of financial investment, resistance from professional associations, prescriptive approaches lacking adaptability and an overly centralised governance model<br>c) The main facilitators were a strong financial commitment using various allocation and payment approaches, the cooperation of professional associations, and an incremental change philosophy based on strong decentralisation of decisions allowing adaption to local circumstances

Study	Country/ Policy/ Methodology	Primary Aim	Type of PC Transformation	Main outcome measurements	Description of Population	Findings
Aggarwal & Williams (2019)[8]	Canada<br><br>Community Health Centres (CHCs); Family Health Networks (FHNs); Family Health Groups (FHGs) & Comprehensive Care Model (CCM); Family Health Teams (FHTs) & Family Health Organizations (FHOs).<br><br>Mixed Methods	To evaluates the pace and direction of primary care reform as well as the extent of resulting change in the organization and delivery of primary care in Ontario.	a) Population health approach<br>b) Group practice setting<br>c) Multi-disciplinary teams<br>d) Alternative payment mechanisms<br>e) Patient enrolment choice<br>f) Community engagement<br>g) 24/7 access to care<br>h) Information technology<br>i) Service coordination and integration<br>j) Continuous performance measurement and quality improvement<br>k) Change in governance	Synthesis of evidence	N/A	a) 11 core dimensions of PC Transformation were identified<br>b) There has been little substantive change in the organisation and delivery of primary care in Ontario over 10 years despite multiple transformation policies.

Study	Country/ Policy/ Methodology	Primary Aim	Type of PC Transformation	Main outcome measurements	Description of Population	Findings
Babiarz et al (2010)[41]	China<br><br>New Rural Cooperative Medical Scheme (NCMS)<br><br>Quantitative	To determine whether the NCMS has affected the operation and use of village health clinics.	a) Alternative payment mechanisms<br>b) Increased financial resources in the system<br>c) Increased primary care access	a) Primary care utilisation<br>b) Out-of-pocket expenditure<br>c) exposure to financial risk	8339 individuals in 160 primary care clinics in 100 villages across 25 rural counties in five provinces.	a) In village clinics NCMS was associated with an increase in weekly patient flow and monthly gross income<br>b) For individuals, participation in NCMS was associated with an increase in village clinic use but no change in overall medical care use. Out of pocket spending and exposure to financial risk decreased<br>c) NCMS partially reduced inequity in rural healthcare

Study	Country/ Policy/ Methodology	Primary Aim	Type of PC Transformation	Main outcome measurements	Description of Population	Findings
Yam et al (2011)[42]	China<br><br>The Elderly Healthcare Voucher Scheme<br><br>Quantitative	To assess whether the voucher scheme, as implemented so far, has reached its intended goals, and how it might be further improved in the context of public-private partnership in Hong Kong	a) Population health approach<br>b) Alternative payment mechanisms<br>c) Financial incentives<br>d) Increased private and/or third sector involvement	Patient views	1,026 people aged 70 or over in Hong Kong	a) 71% of respondents were aware of the voucher scheme but only 35% has used it<br>b) Despite the provision of vouchers valued at US$30 per year as an incentive to encourage the use of private primary care services, after 12-months of implementation, 66.2% of all respondents agreed that they has not changed their health seeking behaviours<br>c) The most common reasons for not utilising the vouchers were familiarity with the public system and the low levels of subsidy<br>d) The policy had not achieved its aim of greater private primary care use.

Study	Country/ Policy/ Methodology	Primary Aim	Type of PC Transformation	Main outcome measurements	Description of Population	Findings
Powell-Jackson et al (2015)[43]	China<br><br>New Cooperative Medical Scheme (NCMS)<br><br>Quantitative	To examine the impact of two key features of healthcare reform on health care utilisation using panel household data.	a) Alternative payment mechanisms<br>b) Financial incentives<br>c) Increased primary care access	a) Primary care utilisation<br>b) Non-primary care healthcare utilisation	6,702 households (30,393 individuals) were interviewed in 260 villages in survey round 1 in 2009. Of these 5,407 households (23,750 individuals) were contacted in the second survey round in 2011. 1,161 new households were added in survey round 2.	a) The redesign of the rural insurance package, in insolation, led to a 47% increase in the use of outpatient care at village clinics and greater intensity of treatment<br>b) The second policy of capitation budget and pay-for-performance showed no effect on health care use over and above that generated by the redesign of the insurance benefit package

Study	Country/ Policy/ Methodology	Primary Aim	Type of PC Transformation	Main outcome measurements	Description of Population	Findings
Ding et al (2016)[44]	China<br><br>National essential medicines policy & Medical insurance system<br><br>Quantitative	To determine the changing prescribing patterns associated with the national essential medicine and medical insurance policies in township hospitals.	a) Alternative payment mechanisms<br>b) Change in governance	Medication use	29 township hospitals from six counties in three provinces of China	a) The average number of medicines and costs per-prescription dropped by ~50%<br>b) Percentage of prescriptions for antibiotics, adrenal corticosteroids, and injections dropped from 54% to 38%, 14% to 4% and 54% to 25% respectively<br>c) Significant regional differences were observed with some underdeveloped regions outperforming more wealthy regions

Study	Country/ Policy/ Methodology	Primary Aim	Type of PC Transformation	Main outcome measurements	Description of Population	Findings
Zhang & Fang (2016)[45]	China<br><br>2009 healthcare reform<br><br>Quantitative	To assess village doctors' job satisfaction during the reforms and to explore factors affecting job satisfaction	a) Population health approach<br>b) Multi-disciplinary teams<br>c) Alternative payment mechanisms<br>d) Financial incentives<br>e) Increased financial resources in the system<br>f) Increased primary care access	GP satisfaction	935 village doctors in Jiangxi Province	a) Only 12.72% of village doctors were either satisfied or very satisfied with their jobs<br>b) The top three items relating to dissatisfaction were: pay and workload, lack of promotion opportunities and work conditions

Study	Country/ Policy/ Methodology	Primary Aim	Type of PC Transformation	Main outcome measurements	Description of Population	Findings
Wong et al (2016)[46]	China<br><br>2009 Health care reform<br><br>Quantitative	To evaluate the effectiveness of China's healthcare reform from 2009 to 2011 by examining China's annual healthcare workforce statistics of 2008 and 2011	a) Population health approach<br>b) Alternative payment mechanisms<br>c) Increased financial resources in the system<br>d) Increased primary care access	Availability of primary healthcare manpower	Area-based statistics from 28 Chinese provinces	a) The 2009 Healthcare reform generally improved access to the primary healthcare workforce in all 28 provinces<br>b) Some provinces managed to develop significantly more than others which widened regional disparity over time

Study	Country/ Policy/ Methodology	Primary Aim	Type of PC Transformation	Main outcome measurements	Description of Population	Findings
Zhang et al (2017)[47]	China<br><br>2009 Healthcare reform<br><br>Quantitative	To examine the changes in efficiency performance and determinants of efficiency after the 2009 reforms to provide evidence to assess the progress of the reform from the perspective of efficiency.	a) Population health approach<br>b) Multi-disciplinary teams<br>c) Alternative payment mechanisms<br>d) Financial incentives<br>e) Increased financial resources in the system<br>f) Increased primary care access	Primary care utilisation	Aggregate statistics from the World bank, the China health statistical yearbook and routine reports 2005-2015	a) Overall efficiency and total factor productivity increased after the reform but, mainly attributed to weakened primary health care, the health care delivery structure showed low system efficiency<br>b) Between 2009 and 2015 the proportion of primary health care workers decreased by 6.8%, the proportion of primary health care outpatient visits decreased by 5.4% and primary health care inpatient care increased by 11.7%

Study	Country/ Policy/ Methodology	Primary Aim	Type of PC Transformation	Main outcome measurements	Description of Population	Findings
Huang et al (2018)[48]	China<br><br>2009 Health care reform<br><br>Quantitative	To examine the availability, use and affordability of medicines in urban China following the 2009 Health care system reform that included the implementation of universal health coverage	Increased primary care access	Medication use	>800 households in Hangzhou and Baoji, China 2009 to 2013	a) The availability of medicines increased in urban areas after the introduction of the reform<br>b) The number of medicines stocked in high income areas decreased, whilst the number stocked in low income areas increased<br>c) Out-of-pocket expenditure on medicines increased for all populations over the study period

Study	Country/ Policy/ Methodology	Primary Aim	Type of PC Transformation	Main outcome measurements	Description of Population	Findings
Zhou et al (2018)[49]	China<br><br>2009 health care reform<br><br>Quantitative	To measure changes in income-related health inequity in China between 2010 and 2014	a) Population health approach<br>b) Multi-disciplinary teams<br>c) Alternative payment mechanisms<br>d) Financial incentives<br>e) Increased financial resources in the system<br>f) Increased primary care access	Self-assessed health	31,743 and 32,006 respondents aged over 15 to the China Family Panel Studies in 2010 and 2014, respectively.	a) From 2010 to 2014, the self-assessed health gap between income groups in China decreased and health equity improved, however health differences remain
Gong et al (2018)[50]	China<br><br>2009 healthcare reform<br><br>Quantitative	To evaluate and compare the quality of community health services before and after the 2009 health care reforms in China.	Increased primary care access	a) Patient satisfaction<br>b) Quality of Healthcare (World Bank framework)	2274 & 2501 community health centres (CHSs), 38,200 & 42,200 prescriptions, and 12,163 & 12,386 outpatients visiting CHCs in 2008 & 2011 respectively were analysed.	a) Quality of healthcare improved following introduction of the 2009 healthcare reform<br>b) Patient satisfaction increased between 2008 and 2011<br>c) Overuse of injectable medicines was not improved by the reform

Study	Country/ Policy/ Methodology	Primary Aim	Type of PC Transformation	Main outcome measurements	Description of Population	Findings
Li et al (2019)[51]	China<br><br>2009 Health Reform<br><br>Quantitative	To partially fill the knowledge gap in understanding of the demand-side changes following the health reform	a) Population health approach<br>b) Alternative payment mechanisms<br>c) Increased primary care access	a) Primary care utilisation<br>b) Non-primary care healthcare utilisation<br>c) Out-of-pocket expenses	2248 & 2121 individuals who responded to the China Health and Retirement Longitudinal Study (CHARLS) in 2008 and 2012 respectively.	a) Older people were more likely to use both outpatient and inpatient care after the reform<br>b) Overall, those with at least one outpatient visit saw a decrease in the ratio of out-of-pocket expenses and pharmaceutical spending to total health expenditure after the reform<br>c) Overall, for those with at least one inpatient stay there was no significant change in the ratio of out-of pocket expenses and pharmaceutical spending to total health expenditure

Study	Country/ Policy/ Methodology	Primary Aim	Type of PC Transformation	Main outcome measurements	Description of Population	Findings
Chen et al (2020)[52]	China<br><br>New Health Care Reform<br><br>Quantitative	To estimate the new health care reform's impact on health insurance coverage and whether an increase in health insurance coverage rate affects health service utilisation efficiency.	a) Increased financial resources in the system<br>b) Increased primary care access	a) Patient views	56,873 respondents to the China Health and Nutrition Survey (CHNS) 1989 to 2015	a) The reform increase health insurance coverage and provided basic universal coverage to all citizens<br>b) This increase in coverage led to increase in healthcare demand<br>c) More people favoured tertiary hospitals for care as opposed to primary care - possibly because they could now afford it and perceive the care to be better

Study	Country/ Policy/ Methodology	Primary Aim	Type of PC Transformation	Main outcome measurements	Description of Population	Findings
Jin et al (2020)[53]	China<br><br>2009 Healthcare reform<br><br>Quantitative	To assess the impact of health insurance and health workforce on healthcare seeking behaviour.	a) Alternative payment mechanisms<br>b) Financial incentives<br>c) Increased primary care access	a) Primary care utilisation<br>b) Non-primary care healthcare utilisation	177,501 respondents from 94 counties and 273,697 respondents from 156 counties to the China National Health Service Surveys in 2008 and 2013 respectively.	a) Increasing health insurance and physician density at primary health care institutions was associated with more outpatient visits and admissions<br>b) Inappropriate hospital use remained high, potentially due to uneven distribution of healthcare resources away from primary health care<br>c) Physician density was higher in county hospitals compared to primary health care institutions

Study	Country/ Policy/ Methodology	Primary Aim	Type of PC Transformation	Main outcome measurements	Description of Population	Findings
Zhou et al (2021)[55]	China<br><br>Hierarchical Medical System (HMS)<br><br>Quantitative	To evaluate the effect of HMS on health seeking behaviour in China using panel data.	a) Alternative payment mechanisms<br>b) Financial incentives<br>c) Increased financial resources in the system<br>d) Increased primary care access	a) Primary care utilisation<br>b) Non-primary care healthcare utilisation	61,932 residents from 25 provinces responding to the China Family Panel Studies 2012, 2014, 2016, and 2018.	a) Implementation of HMS had a significantly positive effect on the probability or urban residents going to primary care facilities, but not significant change was found for rural residents<br>b) Basic health insurance was a significant factor for directing residents to primary care facilities<br>c) The effect of HMS in chronic disease is poor

Study	Country/ Policy/ Methodology	Primary Aim	Type of PC Transformation	Main outcome measurements	Description of Population	Findings
Zhou et al (2014)[56]	China<br><br>2009 Healthcare reform<br><br>Qualitative	To explore the impact of the 2009 reform on healthworkers and service-users at township level, which has been the major target of the first phase of reforms.	a) Population health approach<br>b) Multi-disciplinary teams<br>c) Alternative payment mechanisms<br>d) Financial incentives<br>e) Increased financial resources in the system<br>f) Increased primary care access	a) Patient views<br>b) Other MDT views<br>c) Managerial views	8 health officials, 80 township health workers and 80 service-users in 8 counties in Zhejiang and Yunnan provinces in China.	a) Some elements of the reform may be undermining primary care by contributing to fast-growing medical costs and for an imbalance of benefits between outpatient and inpatient services<br>b) Salary reform has guaranteed health worker's income but greatly reduced their incentives<br>c) The essential drug list removed perverse incentives to overprescribe, but led to falls in income for health workers and loss of autonomy for doctors<br>d) These unintended consequences have led to a brain-drain of experienced health workers from township hospitals and patients have flowed to county hospitals at greater cost.

Study	Country/ Policy/ Methodology	Primary Aim	Type of PC Transformation	Main outcome measurements	Description of Population	Findings
Zhang et al (2017)[57]	China<br><br>2009 Health care reform<br><br>Qualitative	To understand how primary health care providers coped with the new primary health care model and the job characteristics brought about by these changes	a) Population health approach<br>b) Community engagement<br>c) Change in governance<br>d) Increased financial resources in the system<br>e) Increased primary care access	a) GP views<br>b) Other MDT views	30 primary health care providers in Jinan city of Shandong province	a) Primary health care providers employed coping strategies of exit, passive loyalty and compromise to deal with changes in primary health work<br>b) Health workers perceived their jobs as less intensive than hospital work and often more trivial with a heavy workload, blurred job description, unsatisfactory income, and a lack of professional development<br>c) Positive aspect of the role post-reform were having a close relationship with the community they worked in and low work pressure

Study	Country/ Policy/ Methodology	Primary Aim	Type of PC Transformation	Main outcome measurements	Description of Population	Findings
Yuan et al (2019)[58]	China<br><br>Family doctor contracting services<br><br>Qualitative	To identify the facilitators and barriers to implement family doctor contracting services in China.	a) Multi-disciplinary teams<br>b) Alternative payment mechanisms<br>c) Financial incentives<br>d) Increased financial resources in the system	a) GP views<br>b) Other MDT views<br>c) Managerial views	62 policymakers, 19 primary health institution leaders and 48 family doctor team members from 19 primary health institutions in nine provinces from eastern, middle and western areas of China	a) Facilitators for implementing family doctor contracting services included: national reform involving both top-down and bottom-up policy making, financial support, perfomance-based incentives and positive engagement from health administrators<br>b) Barriers included: Lack coordination between departments at national level, distrust in the quality of primary care, lack of government subsidies and health insurance reimbursement and performance ceilings, low competency of family doctors, weak evaluations on performance-based salary and misunderstandings about the policy

Study	Country/ Policy/ Methodology	Primary Aim	Type of PC Transformation	Main outcome measurements	Description of Population	Findings
Wu et al (2016)[59]	China<br><br>No specified policy<br><br>Mixed Methods	To evaluate the effectiveness of a bundled policy on strengthening the country-village communication and improving the quality of chronic disease management	a) Community engagement<br>b) Continuous performance measurement and quality improvement<br>c) Additional training for healthcare practitioners	Chronic disease management	40 administrative staff and public health providers in three counties in Henan Province, China. Administrative health data 2011, 2012, and 2014.	a) There was an increase in follow-up rates and quality markers for hypertension and diabetes in the intervention counties after introduction of the policy<br>b) Training and guidance for staff increased following the intervention meaning more patients were referred to appropriate levels of care
Li et al (2020)[54]	China<br><br>Healthcare management for the aged (HMA)<br><br>Mixed Methods	To understand the achievements made and challenges faced by HMA in Southwest China.	a) Multi-disciplinary teams<br>b) Community engagement	Patient satisfaction<br>b) Patient views<br>c) Other MDT views<br>d) Managerial views	772 survey respondents, 96 older people in 16 focus groups, Interviews with 16 Lay Health Workers (LHWs) and 16 leaders in Primary Healthcare Centres.	a) 94% of survey respondents were satisfied with HMA<br>b) Weakness in HMA design (lack of appropriate assessment indicators, lack of equipment) cited as challenge<br>c) Low capacity and competency of LHWs to deliver HMA and well as insufficient funds and lack of multi-sector cooperation also cited as challenges to implementation of HMA

Study	Country/ Policy/ Methodology	Primary Aim	Type of PC Transformation	Main outcome measurements	Description of Population	Findings
Dourgnon & Naiditch (2010)[60]	France<br><br>Preferred Doctor (PD) scheme<br><br>Quantitative	To describe outcomes one year after PD implementation.	a) Alternative payment mechanisms<br>b) Financial incentives<br>c) Patient enrolment choice<br>d) Information technology	Patient views	7198 individuals from the 2006 French Health, Health Care and Insurance Survey (ESPS)	a) 81% of individuals signed up to the PD scheme with those that: already had a "regular family doctor", already had complementary health insurance, were over 65 or self-declared poor health status being more likely to sign up
Dini et al (2012)[61]	Germany<br><br>Arzt entlastende gemeindenahe E-Health-gestützte Systemische Intervention (AGnES)<br><br>Quantitative	To assess Primary Care Physicians (PCP) overall attitude toward the delegation of home visit tasks, and to determine what they would prefer as the job description and type of employment contract for a qualified Physician Assistant (PA) who would be hired to assist them.	Multi-disciplinary teams	GP views	515 PCPs in three regions of Mecklenburg-Western Pomerania	a) 77% of PCPs were in favour of delegating home visits<br>b) 46% of PCPs were already informally delegating this task<br>c) Female PCPs and those in more rural areas were more likely to favour delegation<br>d) Main advantage of delegation reported was reduced PCP workload and increased PCP satisfaction

Study	Country/ Policy/ Methodology	Primary Aim	Type of PC Transformation	Main outcome measurements	Description of Population	Findings
Tierney et al (2016)[62]	Ireland<br><br>2001 primary care strategy; Primary Care Teams (PCTs)<br><br>Quantitative	To compare primary healthcare professionals' perceptions of the effectiveness of the primary care strategy and PCT implementation in Ireland.	a) Population health approach<br>b) Multi-disciplinary teams<br>c) Community engagement	a) GP views<br>b) Other MDT views	569 GPs and other primary healthcare professionals	a) Respondents across all disciplines agreed interdisciplinary working was important but there had been a lack of progress on implementation<br>b) GPs were more negative about the effectiveness of the strategy compared to other healthcare professionals<br>c) GP participation and resources were the most important factors for effective team working<br>d) Protected time for meetings and capacity to manage workload for meetings were rated as very important factors for effective team working by GPs, clinical therapists and nurses.

Study	Country/ Policy/ Methodology	Primary Aim	Type of PC Transformation	Main outcome measurements	Description of Population	Findings
Tierney et al (2018)[63]	Ireland<br><br>2001 primary care strategy; Primary care teams (PCTs)<br><br>Qualitative	To conduct a theoretically informed, multiperspectival empirical analysis of the implementation of community participation via PCTs in Ireland	a) Multi-disciplinary teams<br>b) Community engagement	a) Patient views<br>b) GP views<br>c) Other MDT views<br>d) Managerial views	39 community representatives, health care professionals policymakers and GPs in focus groups across four case study sites in Ireland	a) There was a lack of clarity and confidence in the role of community representatives in PCTs<br>b) PCTs need to be functioning effectively in order to incorporate community representation<br>c) There was a lack of formal appraisal of the community representative role<br>d) There was general understanding and support of community participation in the case study sites but this was not the true for wider stakeholders and the PCT network

Study	Country/ Policy/ Methodology	Primary Aim	Type of PC Transformation	Main outcome measurements	Description of Population	Findings
McEvoy et al (2019)[64]	Ireland<br><br>The Joint Community Participation in Primary Care Initiative (JI)<br><br>Qualitative	To report a Normalisation Process Theory analysis of the levers and barriers to the implementation of community participation in primary healthcare in Ireland.	Community engagement	a) Patient views<br>b) GP views<br>c) Other MDT views<br>d) Managerial views	Study 1: 33 stakeholders from the Health Service Executive and community organisations.<br>Study 2: 39 community representatives, service planners, and GPs.	a) Community participation in primary care was a new way of working for many stakeholders and they did not always have a clear, shared understanding of the aims, objectives and benefits of the policy<br>b) Policy champions and strong working relationships were integral initiation and implementation<br>c) Funding, organisational support, training and networking beneficial to implementation

Study	Country/ Policy/ Methodology	Primary Aim	Type of PC Transformation	Main outcome measurements	Description of Population	Findings
Tierney et al (2019)[65]	Ireland<br><br>National Primary Care Strategy (2001)<br><br>Mixed Methods	To analyse empirical evidence of "from the ground up" interdisciplinary working in Irish primary care.	a) Population health approach<br>b) Multi-disciplinary teams<br>c) Service coordination and integration	a) GP views<br>b) Other MDT views<br>c) Managerial views	Questionnaire responses from 71 GPs and 498 other healthcare professionals and 37 interviews with 8 GPs, 7 practice managers/admin support and 22 health care professionals in three Health Service Executive regions in Ireland.	a) The majority of respondents agreed that there were other forms of bottom-up interdisciplinary working that were not captured by government metrics<br>b) Coherence among team members was very important to establishing bottom up innovations with informal communication playing a major role<br>c) Levers to enable change include innovations that "make sense" to professionals, based on local needs and focus on preventative patient care<br>d) Bottom up innovations were shaped by the previous top down policy approach by stimulating interactions between health care professionals in primary care about its broad purpose and their respective skill sets.

Study	Country/ Policy/ Methodology	Primary Aim	Type of PC Transformation	Main outcome measurements	Description of Population	Findings
Fiorentini et al (2011)[66]	Italy<br><br>No specified policy<br><br>Quantitative	To estimate the impact of different financial incentives (pay-for-performance, pay-for-participation and pay-for-compliance) on the probability of avoidable hospitalisations	Financial incentives	Non-primary care healthcare utilisation	2,784,099 patients and 3,095 GPs in the Italian region of Emilia-Romagna in 2005	a) Inappropriateness of care was reduced for 27 policy targets identified by the regional health authority but not for internationally classified ACSCs suggesting institutional support is critical to the success of improvement
Buivydiene et al (2010)[67]	Lithuania<br><br>No specified policy<br><br>Quantitative	To evaluate the change in the healthcare indicators in major and minor counties of Lithuania following the healthcare reforms of 2002–5	Increased primary care access	Access to primary care	3 major and 7 minor counties in Lithuania	a) There was an increase in the number of family physicians (FPs) and percent of the population serviced by FPs in both major and minor counties<br><br>b) The number of specialist physicians decreased in major counties but increased in minor counties<br><br>c) The number of nurses increased

Study	Country/ Policy/ Methodology	Primary Aim	Type of PC Transformation	Main outcome measurements	Description of Population	Findings
Van den Hombergh et al (2016)[68]	Netherlands<br><br>2006 Health care reform<br><br>Quantitative	To explore if during the years after 2006 patient experiences of Dutch family practice had changed.	a) Population health approach<br>b) Multi-disciplinary teams<br>c) Alternative payment mechanisms<br>d) Financial incentives<br>e) Increased financial resources in the system<br>f) Additional training for healthcare practitioners	Patient satisfaction	Europep questionnaire 2007-2012. 78,985 patients assessed the performance of 2966 GPs and 45,773 patients assessed the organisation of 1657 practices	The number of patients with positive experiences of their GP and practice organisation increased significantly between 2007 and 2012

Study	Country/ Policy/ Methodology	Primary Aim	Type of PC Transformation	Main outcome measurements	Description of Population	Findings
Matheson et al (2015)[69]	New Zealand<br><br>New Zealand Health Strategy; Primary Health Strategy; District Health Boards; Primary Health Organisations.<br><br>Quantitative	To explore how primary health care policy changes in New Zealand over the last decade have impacted on primary care access equity and avoidable hospital admissions.	Increased primary care access	Non-primary care healthcare utilisation	Hospital discharges from 2001/02 to 2013/14	a) Ambulatory Sensitive hospitalisations (ASH) rates dropped for children over the study period especially those aged 0-4 and from of pacific origin or those living in the most deprived areas<br>b) There was an increase in ASH rates and inequalities for Pacific peoples in the 45-64 age group<br>c) There was no, or very little, change in ASH rates or inequalities for Mãori peoples aged 45-64
Thomson (2018)[70]	New Zealand<br><br>Universal capitated subsidies<br><br>Quantitative	To explore how the distribution of GPs changed in the short and long run from 2002/03 to 2015/16.	a) Population health approach<br>b) Alternative payment mechanisms<br>c) Increased financial resources in the system	Primary care utilisation	12,166, 11,923, and 63,962 respondents to the New Zealand Health Survey (NZHS) from 2002/03, 2006/07 and 2015/16 respectively.	a) Capitation subsidies were associated with improved access to primary care for indigenous Mãori and preventative visits by 2006/07<br>b) However, From 2006/07 onward, patients with the greatest health need began reporting fewer and less frequent doctors' visits per annum

Study	Country/ Policy/ Methodology	Primary Aim	Type of PC Transformation	Main outcome measurements	Description of Population	Findings
Finlayson et al (2012)[71]	New Zealand<br><br>Primary Health Care (PHC) Strategy<br><br>Qualitative	To ascertain how new funding arrangements, introduced in New Zealand's 2001 PHC Strategy, have impacted on the expansion of nurses' role in general practice	a) Multi-disciplinary teams<br>b) Alternative payment mechanisms<br>c) Patient enrolment choice<br>d) Change in governance	a) GP views<br>b) Other MDT views<br>c) Managerial views	128 GPs, Nurses, and healthcare stakeholders.	a) There has been substantial growth in a small number of nurses' roles and capability since the introduction of the PHC strategy particularly regarding chronic disease management and PC access for vulnerable groups<br>b) Nurses are seen as a way to increase income in some GP practices via additional funding for programmes to improve access or tackle inequalities<br>c) At the practice-level, business incentives are still driving many GPs rather than a population-based health approach. This may lead to less use of nurses

Study	Country/ Policy/ Methodology	Primary Aim	Type of PC Transformation	Main outcome measurements	Description of Population	Findings
Ayeleke et al (2020)[72]	New Zealand<br><br>System level measures (SLM) framework<br><br>Qualitative	To explore how the capacity and capability funding has been used and the issues and challenges that have arisen from the funding implementation.	a) Service coordination and integration<br>b) Continuous performance measurement and quality improvement	Key informant views	50 key informants from 18 of New Zealand's 20 districts.	a) The funding for reform was used in three ways: to actively support quality improvement and integration initiatives, to tweak existing performance incentive schemes, and by being passes directly to general practice with no strings attached<br>b) Three issues were identified related to implementation of the reform: lack of clear guidance regarding use of the funding, perception as a barrier to integration, perception finding was insufficient for intended purpose

Study	Country/ Policy/ Methodology	Primary Aim	Type of PC Transformation	Main outcome measurements	Description of Population	Findings
Dimitrovová et al (2020)[73]	Portugal<br><br>Family Health Units (FHUs)<br><br>Quantitative	To evaluate the impact of FHUs implementation on population health outcomes (rate of hospitalisation for ambulatory care sensitive conditions (ACSC)) and to explore the effectiveness of pay-for-performance.	a) Community engagement<br>b) Continuous performance measurement and quality improvement<br>c) Increased primary care access	Non-primary care healthcare utilisation	448 FHUs created in 126 municipalities in Portugal between 2006 and 2015	No significant impact of the FHUs implementation on the reduction of the hospitalisation rate for ASCS was found
da Luz Pereira (2021)[74]	Portugal<br><br>Family Health Units (FHU)<br><br>Quantitative	To evaluate the impact of the introduction of commissioning on indicator results over a period of four years, overall and according to organisational model.	a) Alternative payment mechanisms<br>b) Information technology<br>c) Change in governance	Change in Quality of Care	All mainland primary health care units 2013-2016	a) The average value of most quality of care indicators increased between 2013 and 2016<br>b) Organisational groups with higher functional autonomy and those with pay-for-performance systems appear to perform better than other organisational groups

Study	Country/ Policy/ Methodology	Primary Aim	Type of PC Transformation	Main outcome measurements	Description of Population	Findings
Lapão & Dussault (2012)[75]	Portugal<br><br>Primary health care reform<br><br>Qualitative	To present an initial assessment of the implementation of the primary health care reform and to try to explain the gap between its expected results and what was observed in the field 5 years later.	a) Group practice setting<br>b) Financial incentives<br>c) Change in governance<br>d) Increased primary care access	Managerial views	257 managers from the clinical councils of 73 Health Centre Groups	a) Strengths: Group practice setting offered effective management, good training opportunities, team-work<br>b) Weaknesses: Shortage of staff, low population uptake of family physician, difficult to practice in geographically dispersed populations, Lack of IT, Lack of data, deficient reference system with hospitals<br>c) Opportunities: Partnership with municipal authorities, reorganisation of chronic disease management, IT system development, partnership with universities<br>d) Threats: Difficult communication with regional health authority (ARS), lack of support from ARS, Interference of central services, lack of support from ministry of health.

Study	Country/ Policy/ Methodology	Primary Aim	Type of PC Transformation	Main outcome measurements	Description of Population	Findings
Norwood et al (2019)[76]	Portugal<br><br>Primary health care reform 2005<br><br>Qualitative	To examine patients' views and preferences regarding primary care and the ongoing reform	a) Multi-disciplinary teams<br>b) Alternative payment mechanisms<br>c) Financial incentives	Patient views	69 participants in eight focus groups	a) The majority of participants perceived that the reform was positive but the improvements achieved by the reform were insufficient to lead to most participants having a positive experience of primary care delivery<br>b) Satisfaction/Dissatisfaction with primary care was strongly associated with interpersonal relations and communication with doctors<br>c) Participants valued continuity of care, but felt levels of responsiveness, flexibility and coordination in the current system were still unsatisfactory<br>d) Access ad waiting times were seen as challenging and led participants to seek primary care from emergency departments and private doctors.

Study	Country/ Policy/ Methodology	Primary Aim	Type of PC Transformation	Main outcome measurements	Description of Population	Findings
Isaksson et al (2016)[77]	Sweden<br><br>Primary care choice reform<br><br>Quantitative	To examine how the primary care choice reform has affected geographical equity by analysing patterns of establishment on the part of new private providers.	Increased private and/or third sector involvement	Geographic location of new practices	1411 primary health care centres in Sweden	a) Health centres established after the reform were opened in locations with significantly fewer older adults living alone as well as fewer single parents - groups which generally have lower socioeconomic status and high health care needs<br><br>b) No significant effects were observed for other socioeconomic variables including mean income, percentage of immigrants, education, unemployment and children <5 years

Study	Country/ Policy/ Methodology	Primary Aim	Type of PC Transformation	Main outcome measurements	Description of Population	Findings
Sveréus et al (2018)[78]	Sweden<br><br>Patient choice reform<br><br>Quantitative	To analyse changes in the socioeconomic distribution of GP visits following primary care patient choice reform, and to compare their magnitude and direction in pure capitation, versus capitation/activity-based mixed, provider reimbursement settings.	Increased private and/or third sector involvement	Primary care utilisation	~3.6 million adults over the age of 25 in three Swedish counties.	a) The reform led to increased access to GP visits, but there were only small changes in their socioeconomic distribution<br><br>b) Changes were more pro-poor over time, but it is not clear whether this was at the expense of reduced visit length or content
Dietrichson et al (2019)[79]	Sweden<br><br>Patient Choice Reform<br><br>Quantitative	To identify the effects of increased patient choice and reduced barriers to entry on quality of care.	a) Patient enrolment choice<br><br>b) Increased private and/or third sector involvement	a) Patient satisfaction<br><br>b) Patient views<br><br>c) Non-primary care healthcare utilisation	All primary care centres in Sweden 2005-13 and 30,000-40,000 respondents of the 2009, 2011 & 2013 national patient satisfaction survey.	a) Small improvement in overall patient satisfaction following reform implementation<br><br>b) No significant effects on avoidable hospitalisation or satisfaction with access to care

Study	Country/ Policy/ Methodology	Primary Aim	Type of PC Transformation	Main outcome measurements	Description of Population	Findings
Mosquera et al (2021)[80]	Sweden<br><br>Free choice in primary health care reform<br><br>Quantitative	To evaluate whether the free choice in primary health care reform has impacted on primary health care service performance	a) Patient enrolment choice<br>b) Increased private and/or third sector involvement	Non-primary care healthcare utilisation	Total adult population of the 21 counties of Sweden 2001-2009 (pre-intervention) and 2010-2016 (post-intervention)	a) Following implementation of the reform total hospitalisation rates slowed but acute emergency visits increased<br>b) There was no evidence to support beneficial effects in counties where the reform had been implemented more ambitiously<br>c) Counties with a sustained high presence of private primary care providers displayed the least favourable development related to Ambulatory Care Sensitive Conditions
Avby et al (2019)[81]	Sweden<br><br>National Choice of Care Reform<br><br>Qualitative	To explore what enables primary care innovations	a) Alternative payment mechanisms<br>b) Patient enrolment choice<br>c) Increased private and/or third sector involvement	a) GP views<br>b) Other MDT views<br>c) Managerial views	48 managers and staff at five Primary Health Care Centres (PHCCs) in Jönköping County Council area.	A learning orientated culture including; good leadership, cross-boundary collaboration, visible and understandable performance measures, and ability to adapt all enable innovation in primary care

Study	Country/ Policy/ Methodology	Primary Aim	Type of PC Transformation	Main outcome measurements	Description of Population	Findings
Akhavan & Tillgren (2015)[82]	Sweden<br><br>Care on Equal Terms<br><br>Mixed Methods	To describe and assess client/patient experiences and perceptions of care in four primary health care units (PHCUs) involved in Sweden's national Care on Equal Terms project.	Additional training for healthcare practitioners	Patient views	21 clients/patients and three patient association representatives from four of seven PHCUs participating in Equal Terms project. Response rate to national survey 37.2%-53.4% in participating PHCUs.	a) Perceived quality of care improved in two out of four PHCUs<br>b) Health care providers perception of ethnic origin and mental health status were reported to be important to achieve equitable health care<br>c) Participants reported longer consultations, longer opening hours and better communication as ways to improve health care equity
Burström et al (2017)[6]	Sweden<br><br>Primary Health Care (PHC) Choice Reform<br><br>Literature review	To present the findings of a review of the existing evidence of the impacts of the PHC Health Choice Reform	a) Patient enrolment choice<br>b) Increased private and/or third sector involvement	Synthesis of evidence	6 scientific articles and 9 grey literature reports were included	a) The policy resulted in an increase in the number of visits in PC - particularly for those from affluent groups and those with lower health care needs<br>b) Resource allocation is less dependent on need and more dependent on location, patient choice and demand<br>c) Equity in primary care has reduced as a result of the policy

Study	Country/ Policy/ Methodology	Primary Aim	Type of PC Transformation	Main outcome measurements	Description of Population	Findings
Hone et al (2016)[83]	Turkey<br><br>Family Medicine Model (FMM)<br><br>Quantitative	To evaluate the effect of the new FM model on service utilisation and user satisfaction	a) Multi-disciplinary teams<br>b) Alternative payment mechanisms<br>c) Financial incentives<br>d) Patient enrolment choice<br>e) Increased financial resources in the system<br>f) Increased primary care access	a) Patient satisfaction<br>b) Primary care utilisation<br>c) Non-primary care healthcare utilisation	56,232 respondents to the Life Satisfaction Survey over 8 years 2005-12 (range 6,442-7956)	a) FMM introduction was associated with increased primary care consultations per person and slower growth in primary health care and secondary care consultations<br>b) Patient satisfaction increased after the introduction of FMM<br>c) Negative characteristics of health care service (poor facility hygiene, difficulty getting an appointment, poor physician behaviour and cost) all declined but remained high in urban, low-income, and working age populations

Study	Country/ Policy/ Methodology	Primary Aim	Type of PC Transformation	Main outcome measurements	Description of Population	Findings
Sparkes et al (2019)[84]	Turkey<br><br>Family Medicine Model (FMM)<br><br>Quantitative	To establish the impact of the introduction of the Family Medicine Model patient satisfaction in the Turkish health system.	a) Alternative payment mechanisms<br>b) Patient enrolment choice<br>c) Increased financial resources in the system<br>d) Additional training for healthcare practitioners<br>e) Increased primary care access	Patient satisfaction	69,028 primary health care patients across 81 provinces in Turkey	The FMM significantly improved patient satisfaction with clinical behaviour and organisation of care

Study	Country/ Policy/ Methodology	Primary Aim	Type of PC Transformation	Main outcome measurements	Description of Population	Findings
Erus (2019)[85]	Turkey<br><br>Family Medicine<br><br>Quantitative	To measure the impact of increased health insurance coverage and the family medicine system on out-of-pocket expenses	a) Alternative payment mechanisms<br>b) Financial incentives<br>c) Patient enrolment choice<br>d) Increased financial resources in the system<br>e)Increased primary care access	Out-of-pocket expenses	~26,000 (2003) and ~8,000-10,000 (2008, 2010 & 2013) respondents to the Household Budget Survey of Turkey.	a) After introduction of the policies, increase in OOP expenditures was experience by low-income households but not higher income households<br>b) This did not result in a decrease in access to services - primary care was easier to access<br>c) There was not a decrease in expensive secondary and tertiary care services
Cevik & Kilic (2017)[86]	Turkey<br><br>Family Physician (FP) approach<br><br>Qualitative	To evaluate opinions of patients, health care workers, and managers.	a) Multi-disciplinary teams<br>b) Patient enrolment choice	a) Patient views<br>b) GP views<br>c) Other MDT views<br>d) Managerial views	6 health managers, 16 health providers and 16 patients in one Turkish province	a) Health care professionals and patients felt physician care was superior after the policy implementation<br>b) Primary care facilities were also better following FP implementation<br>c) Facets of primary care that were better before the policy included; family planning and infectious disease surveillance

Study	Country/ Policy/ Methodology	Primary Aim	Type of PC Transformation	Main outcome measurements	Description of Population	Findings
Espinosa-González & Normand (2019)[87]	Turkey<br><br>Family Medicine Programme (FMP)<br><br>Qualitative	To identify the challenges and limitations that primary health care physicians and academicians have encountered in the implementation of the FMP which could have influenced the delivery of care and utilisation	a) Multi-disciplinary teams<br>b) Alternative payment mechanisms<br>c) Financial incentives<br>d) Information technology	a) GP views<br>b) Academicians views	Seven physicians and eight academicians	a) Poor planning, inadequate political commitment to integration of primary health care in the overall system, and collateral effects of a market model in healthcare emerged as limitations to FMP implementation<br>b) Minor themes identified included: uncertainty about the quality of care and physicians' ethical values as well as perceptions of organisational injustice among health care workers were also identified as challenges

Study	Country/ Policy/ Methodology	Primary Aim	Type of PC Transformation	Main outcome measurements	Description of Population	Findings
Kontopantelis et al (2015)[88]	UK<br><br>Quality and Outcomes Framework (QOF)<br><br>Quantitative	To quantify the relationship between the UK's QOF, and all-cause and cause-specific premature mortality linked closely with conditions included in the framework	a) Alternative payment mechanisms<br>b) Financial incentives	a) All-cause mortality<br>b) Cause-specific mortality	8647 English general practices in 32,482 Lower Super Output Areas (LSOAs)(geographical neighbourhoods ~1500 people) in England	a) There were no significant relationships between QOF indicators and all-cause or cause-specific mortality<br>b) All-cause and cause-specific mortality rates declined over the study period<br>c) Higher mortality was associated with greater area deprivation, urban location, and higher proportion of a non-white population
Ryan et al (2016)[89]	UK<br><br>Quality and Outcomes Framework (QOF)<br><br>Quantitative	To test whether QOF was associated with reduced population mortality	a) Financial incentives<br>b) Continuous performance measurement and quality improvement	Cause-specific mortality	Country-level, cause-specific mortality and population data from the WHO morality database between 1994 and 2010.	QOF was not associated with changes on population mortality for a composite outcome, ischaemic heart disease, cancer, or all other non-QOF-targeted conditions

Study	Country/ Policy/ Methodology	Primary Aim	Type of PC Transformation	Main outcome measurements	Description of Population	Findings
Moran et al (2017)[90]	UK<br><br>Clinical Commissioning Groups (CCGs)<br><br>Quantitative	To explore GP attitudes to involvement in commissioning and purchasing of services and future intentions for engagement	a) Multi-disciplinary teams<br>b) Alternative payment mechanisms<br>c) Service coordination and integration<br>d) Change in governance	GP views	2611 GPs responding to the eighth national GP work life survey in 2015	a) Only a minority of GPs felt that commissioning was an important part of their role<br>b) Few of those not currently in leadership roles planned to take up such a role in future. Many current leaders intended to quit before 2020<br>c) Only a minority of respondents felt "ownership" of their local CCG

Study	Country/ Policy/ Methodology	Primary Aim	Type of PC Transformation	Main outcome measurements	Description of Population	Findings
Lopez-Bernal et al (2017)[91]	UK<br><br>Clinical Commissioning Groups (CCGs)<br><br>Quantitative	To assess the association between the NHS reforms and hospital admissions and out-patient specialist visits.	a) Alternative payment mechanisms<br>b) Change in governance	Non-primary care healthcare utilisation	Quarterly hospital admission data in England and Scotland April 2007 to December 2015	a) Giving control of healthcare budgets to GP-led CCGs was not associated with a reduction in overall hospitalisations and was associated with an increase in specialist visits<br>b) After CCGs were introduced there was an increase in total and GP-referred specialist visits equivalent to 12.7% and 19.1% more visits per quarter by the end of 2015<br>c) There was no change in the trend in hospitalisations over the study period
Tames et al (2019)[92]	UK<br><br>Named GP scheme<br><br>Quantitative	To investigate whether the introduction of a named general practitioner improved patients' healthcare for patients aged 75 and over in England	Continuous performance measurement and quality improvement	a) Non-primary care healthcare utilisation<br>b) Continuity of care	Random sample of 27,500 patients aged 65 to 84 in 2012 within 139 English practices from the Clinical Practice Research Datalink (CPRD)	a) The intervention was associated with a decrease in continuity index scores<br>b) The probability of an emergency hospital admission increased after the intervention<br>c) The average number of emergency hospital admissions increased after the intervention

Study	Country/ Policy/ Methodology	Primary Aim	Type of PC Transformation	Main outcome measurements	Description of Population	Findings
Forbes et al (2020)[93]	UK<br><br>No specified policy<br><br>Quantitative	To explore whether increasing the size of the practice population and working collaboratively are linked to changes in continuity of care or access to care	a) Group practice setting<br>b) Multi-disciplinary teams<br>c) Service coordination and integration	Patient views	7089 GP practices in England	a) Larger general practice size may be associated with poorer continuity of care and may not improve patient access<br>b) Close collaborative working did not have any demonstrable effect on patient experience
Gridley et al (2012)[94]	UK<br><br>Clinical Commissioning Groups (CCGs)<br><br>Qualitative	To explore some of the key assumptions underpinning the continued development of general practitioner-led commissioning in health services	a) Group practice setting<br>b) Service coordination and integration<br>c) Increased private and/or third sector involvement	Synthesis of evidence	187 professionals and 99 people affected by services in 10 difference Primary Care Trusts	a) General Practitioners do not always have a pivotal role for all patients e.g. those with neurological conditions managed by specialist teams/nurses. Therefore not ideally placed to commission services<br>b) Little change can be enacted in the absence of centrally set performance managed targets - even if they are unpopular<br>c) The lack of centrally set targets is also likely to lead to geographical inequalities

Study	Country/ Policy/ Methodology	Primary Aim	Type of PC Transformation	Main outcome measurements	Description of Population	Findings
O'Donnell et al (2012)[95]	UK<br><br>Keep Well<br><br>Qualitative	To explore the issues and tensions underpinning the implementation of national programme of anticipatory care	a) Population health approach<br>b) Service coordination and integration	a) GP views<br>b) Other MDT views<br>c) Managerial views	74 individuals were interviewed over 3 years generating a total of 118 interviews.	a) The key practitioner in many GP practices related to the programme was not the GP but the practice nurse who conducted health checks and referred to other practitioners<br>b) Service coordination and integration is often dependent on previous relationships and collaborations within a geographical area<br>c) Population health approach has significant workload and resource implications which require ongoing support to continue development

Study	Country/ Policy/ Methodology	Primary Aim	Type of PC Transformation	Main outcome measurements	Description of Population	Findings
Allan et al (2014)[96]	UK<br><br>not specified<br><br>Qualitative	To explore the experiences of governance and incentives during organizational change for managers and clinical staff	a) Multi-disciplinary teams<br>b) Financial incentives<br>c) Service coordination and integration<br>d) Change in governance	a) Patient views<br>b) GP views<br>c) Other MDT views<br>d) Managerial views	32 service users, 32 managers, and 56 frontline staff in three sites in England	a) Staff were uncertain about their new roles and responsibilities<br>b) Staff felt the organisational change was "top-down" and were unable to influence policy<br>c) Managers tended to focus on service restructure and finances whilst staff focussed on meeting service users' expectations<br>d) Both managers and staff struggled to make sense of policy language.

Study	Country/ Policy/ Methodology	Primary Aim	Type of PC Transformation	Main outcome measurements	Description of Population	Findings
Petsoulas et al (2014)[97]	UK<br><br>Clinical Commissioning Groups (CCGs)<br><br>Qualitative	To explore the attitudes of CCGs towards outsourcing commissioning support functions during the initial stage of the reform.	a) Alternative payment mechanisms<br>b) Service coordination and integration<br>c) Change in governance<br>d) Increased private and/or third sector involvement	a) GP views<br>b) Other MDT views<br>c) Managerial views	96 interviews and observation of 146 meetings	a) Many CCGs were reluctant to outsource core commissioning support functions for fear of losing local knowledge and trusted relationships<br>b) Others reported poor choice in outsourcing options<br>c) Many CCGs expressed doubts about the expectation the outsourcing of commissioning support functions would result in lower administrative costs

Study	Country/ Policy/ Methodology	Primary Aim	Type of PC Transformation	Main outcome measurements	Description of Population	Findings
Humphrey & Cleaver (2018)[98]	UK<br><br>Five year forward view<br><br>Qualitative	To produce a description of how GPs construct their current and future general practice, professional status and identify within the context of the current NHS transformation agenda.	a) Multi-disciplinary teams<br>b) Alternative payment mechanisms; Community engagement<br>c) 24/7 access to care<br>d) Service coordination and integration<br>e) Change in governance	GP views	10 GPs working across three clinical commissioning groups in South East England.	a) GPs were both willing and reluctant to adopt their new roles<br>b) GPs struggled with inter-organisational and cultural barriers and their changing personal identity<br>c) There was a consensus view among participants that expansion of MDT might lead to a reduction in continuity of care
Checkland et al (2013)[99]	UK<br><br>Clinical Commissioning Groups (CCGs)<br><br>Mixed Methods	To explore the development of CCGs in the context of what is known from previous studies of GP involvement in commissioning.	Change in governance	Synthesis of evidence	96 interviews with CCG staff, 146 meetings observed	a) Findings from early stages of CCG implementation suggest still some uncertainty of the role of CCGs

Study	Country/ Policy/ Methodology	Primary Aim	Type of PC Transformation	Main outcome measurements	Description of Population	Findings
Checkland et al (2016)[100]	UK<br><br>Clinical Commissioning Groups (CCGs)<br><br>Mixed Methods	To offer evidence about how the establishment of CCGs played out in practice and examine the implications of the complexity and variation which emerged	Change in governance	Synthesis of evidence	Phase 1: (2011-12): 96 interviews with wide variety of CCG staff and lay members, meeting observation (439 hours), 222 respondents to two online surveys and 38 follow-up interviews with CCG leaders. Phase 2: (2013-15): 42 interviews with GPs and managers. Observation of 48 meetings.	a) CCGs are heterogenous in terms of size, structure, and how functions are distributed between bodies and GP roles<br>b) The lack of formal structure makes understanding accountability at different levels difficult

Study	Country/ Policy/ Methodology	Primary Aim	Type of PC Transformation	Main outcome measurements	Description of Population	Findings
McDermott et al (2019)[101]	UK<br><br>Clinical Commissioning Groups (CCGs)<br><br>Mixed Methods	To analyse how CCGs have responded to the new responsibility to commission primary care services and to identify challenges and factors that facilitated or inhibited achievement of integrated care systems	a) Group practice setting<br>b) Alternative payment mechanisms<br>c) Service coordination and integration<br>d) Change in governance	GP views; Managerial views	147 CCG application documents, two telephone surveys with 49 & 21 CCGs, 48 interviews with CCG policymakers and staff, observation of 74 primary care commissioning meetings and their subgroups (~111 hours)	a) CCGs were using their commissioning power to support group working between primary care practices, but there was limited evidence of wider integration of care with social or secondary care services<br>b) CCGs had a clear focus on maintaining the sustainability of primary care<br>c) There was concern from primary care stakeholders that further integration with non-primary care services could result in reduction in funding for primary care

Study	Country/ Policy/ Methodology	Primary Aim	Type of PC Transformation	Main outcome measurements	Description of Population	Findings
Boeckxstaens et al (2011)[102]	UK<br><br>Quality and Outcomes Framework (QOF)<br><br>Literature review	To describe the evolution of pre-existing (in)equity in health care in the period after the introduction of the QOF in the UK and to describe (in)equities in exception reporting.	a) Alternative payment mechanisms<br>b) Financial incentives	Synthesis of evidence	27 included studies	a) None of the included studies assessed equity in access to health care<br>b) All citizens tend to benefit from QOF but this depends on the individual study<br>c) In general, the introduction of QOF was favourable to the aged and for males<br>d) QOF scores did not vary according to ethnicity. Small differences were found which favoured less deprived groups.

Study	Country/ Policy/ Methodology	Primary Aim	Type of PC Transformation	Main outcome measurements	Description of Population	Findings
Gabbay et al (2011)[103]	USA<br><br>Patient centred medical home (PCMH) and Chronic care model (CCM)<br><br>Quantitative	To describe a major multistakeholder, multipayer inititative to implement the CCM and PCMH across the state of Pennsylvania	a) Multi-disciplinary teams<br>b) Community engagement<br>c) Information technology<br>d) Service coordination and integration<br>e) Continuous performance measurement and quality improvement	Clinical indicators relating to Diabetes care	10,016 patients with diabetes in four regional collaboratives across Pennsylvania in 2008-09	a) There was significant improvement in the percentage of patients who had evidence-based complications screening and who were on therapies to reduce morbidity and mortality<br>b) There was small but statistically significant improvements in key clinical parameters for blood pressure and cholesterol levels, with the greatest absolute improvement in the highest-risk patients
Kuo et al (2013)[104]	USA<br><br>No specified policy<br><br>Quantitative	To assess the impact of state regulations on the increase in care provided by Nurse Practitioners (NPs) in the United States	Multi-disciplinary teams	Patients receiving care from Nurse Practitioners	5% sample of Medicare beneficiaries in the United States	a) Between 1998 and 2010 the number of Medicare patients receiving care from NPs increased fifteen-fold<br>b) States with the least restrictive regulations of NP practice had a 2.5 fold greater likelihood of patients' receiving their primary care from NPs than did the most restrictive states

Study	Country/ Policy/ Methodology	Primary Aim	Type of PC Transformation	Main outcome measurements	Description of Population	Findings
Phillips Jr et al (2014)[105]	USA<br><br>Illinois Health Connect (IHC); Your Healthcare Plus (YHP)<br><br>Quantitative	To explore outcomes associated with IHC and YHP.	a) Population health approach<br>b) Alternative payment mechanisms<br>c) Financial incentives<br>d) Information technology<br>e) Service coordination and integration<br>f) Continuous performance measurement and quality improvement	a) Primary care utilisation<br>b) Non-primary care healthcare utilisation<br>c) Quality of care	>2,000,000 people in the Illinois Medicaid claims and eligibility data from 2004 to 2010.	a) There were annual savings of 6.5% for IHC and 8.6% for YHP by the fourth year, with cumulative Medicaid savings of $1.46 billion. Per-beneficiary annual costs fell in Illinois over this period compared with those in states with similar Medicaid programs<br>b) Quality improved for nearly all metrics under IHC, and most prevention measures more than doubled in frequency<br>c) Medicaid inpatient costs fell by 30.3% and outpatient costs rose by 24.9% across IHC & YHP. Avoidable hospitalisations fell by 16.8% for YHP and bed-days fell by 15.6% for IHC. Emergency department visits declined by 5%

Study	Country/ Policy/ Methodology	Primary Aim	Type of PC Transformation	Main outcome measurements	Description of Population	Findings
Lemak et al (2015)[106]	USA<br><br>Physician Group Incentive Program<br><br>Quantitative	To report on an independent analysis of the impact of the Blue Cross Blue Shield of Michigan's Physician Group Incentive Program on health care spending and quality.	a) Alternative payment mechanisms<br>b) Financial incentives	Healthcare spending and quality of care	>3.2 million people under 65 years of age during 2008-11 for cost analysis and 2008-10 for quality analysis	a) Participation in the incentive program was associated with lower total spending and equivalent or better performance on eleven of fourteen quality indicators over time
Kern et al (2016)[107]	USA<br><br>Patient-Centred Medical Home (PCMH)<br><br>Quantitative	To determine effects of the PCMH on health care quality and utilisation compared with paper records alone and EHRs alone, with extended follow-up.	a) Group practice setting<br>b) Multi-disciplinary teams<br>c) Alternative payment mechanisms<br>d) Information technology<br>e) Service coordination and integration	a) Primary care utilisation<br>b) Non-primary care healthcare utilisation<br>c) Quality of care	438 primary care physicians in 226 practices with 136,480 patients 2008 to 2012	a) Patterns of quality were similar across PCMH, EHR-only and paper-only groups<br>b) Hospitalisations decreased in the PCMH group in 2012<br>c) Emergency Department visits were highest in the PCMH group and lowest in the paper-only group but the rate of change did not differ across groups

Study	Country/ Policy/ Methodology	Primary Aim	Type of PC Transformation	Main outcome measurements	Description of Population	Findings
Shi et al (2017)[108]	USA<br><br>Patient-centred medical home (PCMH)<br><br>Quantitative	To evaluate the relationship between PCMH recognition in Health Centres (HCs) and clinical performance	a) Alternative payment mechanisms<br>b) Information technology<br>c) Continuous performance measurement and quality improvement<br>d) Change in governance<br>e) Increased primary care access	Quality of care indicators	Data from 1,087 HCs in the 2012 Uniform Data System	HCs that had gained PCMH recognition generally performed better on clinical measures than those without PCMH recognition

Study	Country/ Policy/ Methodology	Primary Aim	Type of PC Transformation	Main outcome measurements	Description of Population	Findings
Rhodes et al (2017)[109]	USA<br><br>The Patient Protection and Affordable Care Act (ACA)<br><br>Quantitative	To identify changes in the proportion of primary care providers with appointment availability before and after the major ACA insurance expansions in late 2012/early 2013 and mid-2014 respectively.	Increased primary care access	Primary care appointment availability	Simulated appointment calls to 5,385 private insurance, and 4,352 Medicaid practices in 10 US states in 2012/13 and 2014.	a) Overall appointment rates for private insurance remained stable when comparing Winter 2012/13 to Summer 2014. Pennsylvania and Massachusetts saw significant increases<br>b) Overall, Medicaid appointment rates increased with substantial variation across states<br>c) There was no statistically significant change in wait times when comparing the two periods
Vest et al (2018)[110]	USA<br><br>Wraparound services<br><br>Quantitative	To determine whether the provision of wraparound services within the primary care setting of a large urban safety-net provider was effective in reducing costly health care	a) Population health approach<br>b) Multi-disciplinary teams<br>c) Community engagement	Non-primary care healthcare utilisation	14,096 adult patients who received care from Eskenazi Health in Indiana in the period 2006-16 who had at least one primary care visit before and after 2011 and had received at least one wraparound service.	Receipt of wraparound services consisting of nonmedical interventions that directly addressed the social determinants of health was associated with a reduction in the expected number of subsequent hospitalisations and emergency department visitis

Study	Country/ Policy/ Methodology	Primary Aim	Type of PC Transformation	Main outcome measurements	Description of Population	Findings
Wong et al (2018)[111]	USA<br><br>Massachusetts Health Reform (MHR)<br><br>Quantitative	To examine whether MHR was associated with changes in Veteran's Affairs (VA) Primary Care (PC) use.	a) Increased private and/or third sector involvement<br>b) Other: Mandated minimum level of health insurance, subsidies for low-income households<br>c) Other: Medicaid expansion	Primary care utilisation	147,836 veterans residing in Massachusetts and New England.	a) PC use was not significantly different between Massachusetts and New England following MHR for non-Medicaid enrolled veterans<br>b) MHR was associated with an increase in PC use for VA-Medicaid dual enrollees<br>c) VA enrollees continued to rely on VA PC despite implementation of MHR

Study	Country/ Policy/ Methodology	Primary Aim	Type of PC Transformation	Main outcome measurements	Description of Population	Findings
Jones et al (2018)[112]	USA<br><br>Homeless patient aligned care teams (HPACTs)<br><br>Quantitative	To assess differences in primary care experiences between homeless and nonhomeless Veterans receiving care in Veterans Health Administration (VHA) facilities that had HPACTs available and in VHA facilities lacking HPACTs	a) Community engagement<br>b) Service coordination and integration<br>c) Additional training for healthcare practitioners<br>d) Increased primary care access	Patient views	319,927 (3.2% homeless) respondents from non-HPACT facilities and 22,963 (8.8% homeless) respondents from HPACT facilities to the Patient-Centred Medical Home Survey of Healthcare Experiences (PCMH-SHEP) in 2014-15	VHA facilities with HPACT programmes appear to offer a better primary care experience for homeless versus nonhomeless Veterans
Bustamante & Chen (2018)[113]	USA<br><br>Affordable Care Act (ACA)<br><br>Quantitative	To examine short-term changes in perceived barriers to access to primary care before and after implementations of the ACA among adults in the USA	a) Increased financial resources in the system<br>b) Increased primary care access	Patient views	~23,000 respondents per year to the National Health Interview Survey (NIHS) 2011-2014	a) Individuals were progressively less likely to report challenges to accessing primary care following implementation of ACA<br>b) Individuals were less likely to report inconveniences linked to waiting times for an appointment

Study	Country/ Policy/ Methodology	Primary Aim	Type of PC Transformation	Main outcome measurements	Description of Population	Findings
Singh & Wilk (2019)[114]	USA<br><br>Affordable Care Act (ACA)<br><br>Quantitative	To examine how Medicaid expansions affected access to primary care, and how race/ethnicity moderated these effects.	Increased primary care access	Access to primary care	65,697 respondents to the Behavioural Risk Factor Surveillance System (BRFSS) survey.	a) On average, adults in states with Medicaid expansion were more likely to have insurance and usual source of care and less likely to delay care due to cost post-expansion<br><br>b) Gains in access to care were similar across racial groups such that, in absolute terms, racial/ethnic disparities in access to care persisted even after Medicaid expansion went into effect<br><br>c) One exception was where adult Hispanics had a lower likelihood of having insurance post-expansion

Study	Country/ Policy/ Methodology	Primary Aim	Type of PC Transformation	Main outcome measurements	Description of Population	Findings
Rittenhouse et al (2020)[115]	USA<br><br>Patient-centred medical home (PCMH)<br><br>Quantitative	To provide a perspective on the implementation of PCMH and health IT elements in a variety of US physician practices	a) Multi-disciplinary teams<br>b) Alternative payment mechanisms<br>c) Financial incentives<br>d) Information technology<br>e) Service coordination and integration<br>f) Continuous performance measurement and quality improvement	PCMH adoption	2,532 practices responding to the 2018 American board of family medicine continuous certification exam registration questionnaire.	a) There has been substantial progress in implementing PCMH, but there are variations across practice ownership type<br>b) Federally qualified health centres and Federal military practices were most likely to have adopted PCMH elements. Independently owned practices (one-third of primary care delivery) and rural health clinics were less likely<br>c) Large practice size was also associated with adoption of PCMH elements

Study	Country/ Policy/ Methodology	Primary Aim	Type of PC Transformation	Main outcome measurements	Description of Population	Findings
Brown et al (2021)[116]	USA<br><br>Affordable Care Act (ACA) Medicaid expansion<br><br>Quantitative	To measure the impact of ACA Medicaid expansion on preventable hospitalization (PH) rates, a measure of access to primary care.	a) Continuous performance measurement and quality improvement<br>b) Increased primary care access	a) Access to primary care<br>b) Non-primary care healthcare utilisation	2,103,114 preventable hospitalisations affecting an unreported number of individuals aged 18-65 in eight states.	a) There was no significant change in PHs for states that expanded Medicaid<br>b) Increase in state unemployment rate is associated with an increase in PHs<br>c) Increased Medicaid spending per enrollee and income eligibility were associated with reduced PHs

Study	Country/ Policy/ Methodology	Primary Aim	Type of PC Transformation	Main outcome measurements	Description of Population	Findings
Quinn et al (2013)[117]	USA<br><br>Patient Centred Medical Home (PCMH)<br><br>Qualitative	To characterise early PCMH adoption experiences at safety net health centres (SNHCs)	a) Multi-disciplinary teams<br>b) Alternative payment mechanisms<br>c) Financial incentives<br>d) Information technology<br>e) Continuous performance measurement and quality improvement<br>f) Increased primary care access	a) GP views<br>b) Other MDT views<br>c) Managerial views	98 administrators, providers and clinical staff at 20 of 65 SNHCs in five states.	a) Participants anticipated an improvement in staff satisfaction and patient care and outcomes as a result of transitioning to PCMH<br>b) Obstacles to implementation included staff resistance and lack of financial support<br>c) Enablers to implementation were inclusion of all members of staff, and using data for feedback

Study	Country/ Policy/ Methodology	Primary Aim	Type of PC Transformation	Main outcome measurements	Description of Population	Findings
Dill et al (2021)[118]	USA<br><br>Patient centred medical home (PCMH)<br><br>Qualitative	To address the gap in knowledge in best practices for staffing, human resource policies and practices, and training of both new skills and new roles such as medical assistants (MAs) in PCMH models in four large US health care systems.	a) Multi-disciplinary teams<br>b) Additional training for healthcare practitioners	a) GP views<br>b) Other MDT views<br>c) Managerial views	115 key informants including GPs, physician assistants, nurse practitioners, nurses, medical assistants, program managers, educators and other key stakeholders.	a) Local labour market is very influential on the ability to hire and retain skilled Mas<br>b) Although larger health care organisations have more resources for training and increased compensation, training for MAs was inconsistent across primary care clinics<br>c) Burnout and turnover among MAs as they took on new roles and responsibilities required attention

Study	Country/ Policy/ Methodology	Primary Aim	Type of PC Transformation	Main outcome measurements	Description of Population	Findings
Bidassie et al (2014)[119]	USA<br><br>Patient-Centered Medical Home (PCMH); Patient Aligned Care Team (PACT) Collaborative<br><br>Mixed Methods	To describe and examine VHA's experience disseminating PACT transformation using a Breakthrough Series Collaborative method	a) Multi-disciplinary teams<br>b) Continuous performance measurement and quality improvement<br>c) Change in governance<br>d) Increased primary care access	a) GP views<br>b) Other MDT views<br>c) Managerial views	250-350 individuals from 141 teams participating in face-to-face learning sessions.	a) The PACT collaborative intervention was perceived to be necessary in order to implement PACT in practice<br>b) More than three learning sessions may be required in order to disseminate the PACT model through primary care

Study	Country/ Policy/ Methodology	Primary Aim	Type of PC Transformation	Main outcome measurements	Description of Population	Findings
Peikes et al (2018)[120]	USA<br><br>Comprehensive Primary Care Initiative (CPC)<br><br>Mixed Methods	To evaluate CPC effects on care delivery and outcomes for fee-for-service Medicare beneficiaries attributed to initiative practices, relative to those attributed to matched comparison practices.	a) Population health approach<br>b) Financial incentives<br>c) Community engagement<br>d) Service coordination and integration<br>e) Continuous performance measurement and quality improvement<br>f) Increased primary care access	a) GP views<br>b) Other MDT views<br>c) Managerial views<br>d) Primary care utilisation<br>e) Non-primary care healthcare utilisation<br>f) Healthcare spending, quality of care	497 primary care practices participating in CPC and 908 comparison practices	a) CPC practices reported improvements in primary care delivery, including care management for high-risk patients, enhanced access, and improved coordination of care transitions<br>b) CPC practices showed a reduction in the growth of emergency department visits<br>c) Medicare spending did not reduce by enough to cover care management fees<br>d) CPC did not improve physician or beneficiary experience or practice performance on a limited set of claims-based quality measures.

Figure S1: Included studies by country and methodology

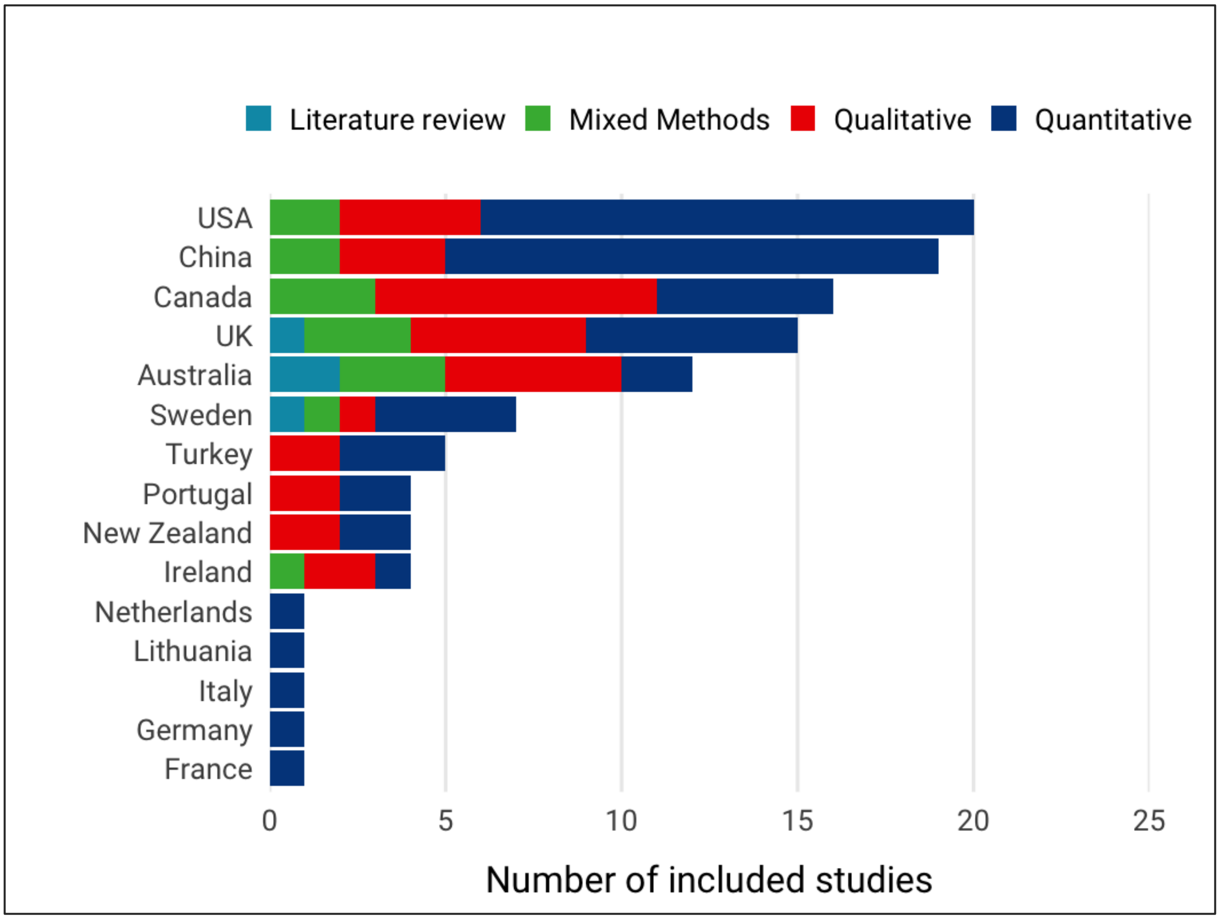

Figure S2: Count of types of primary care transformation in included studies, by policy

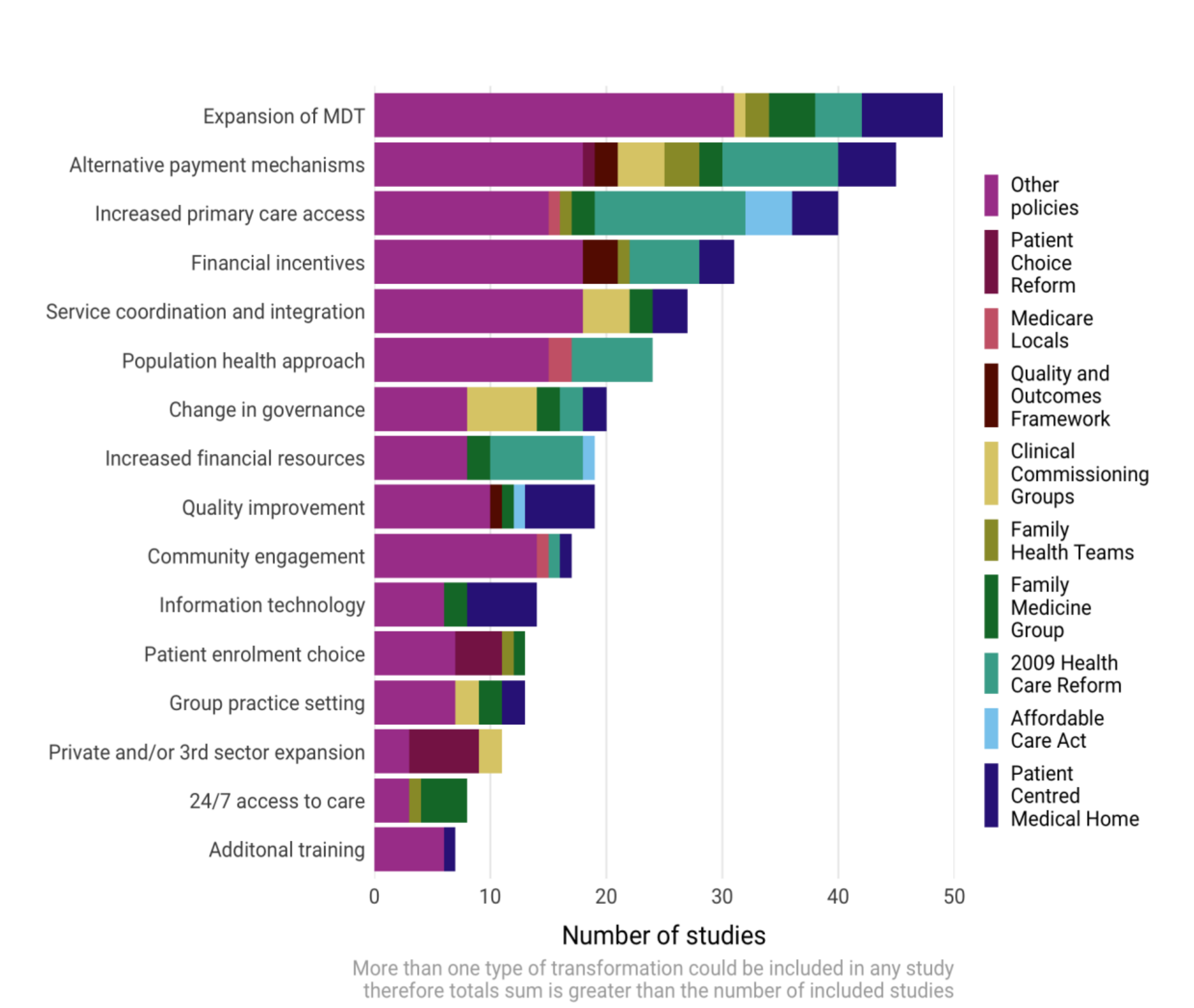

Figure S3: Count of types of primary care transformation in included studies, by policy

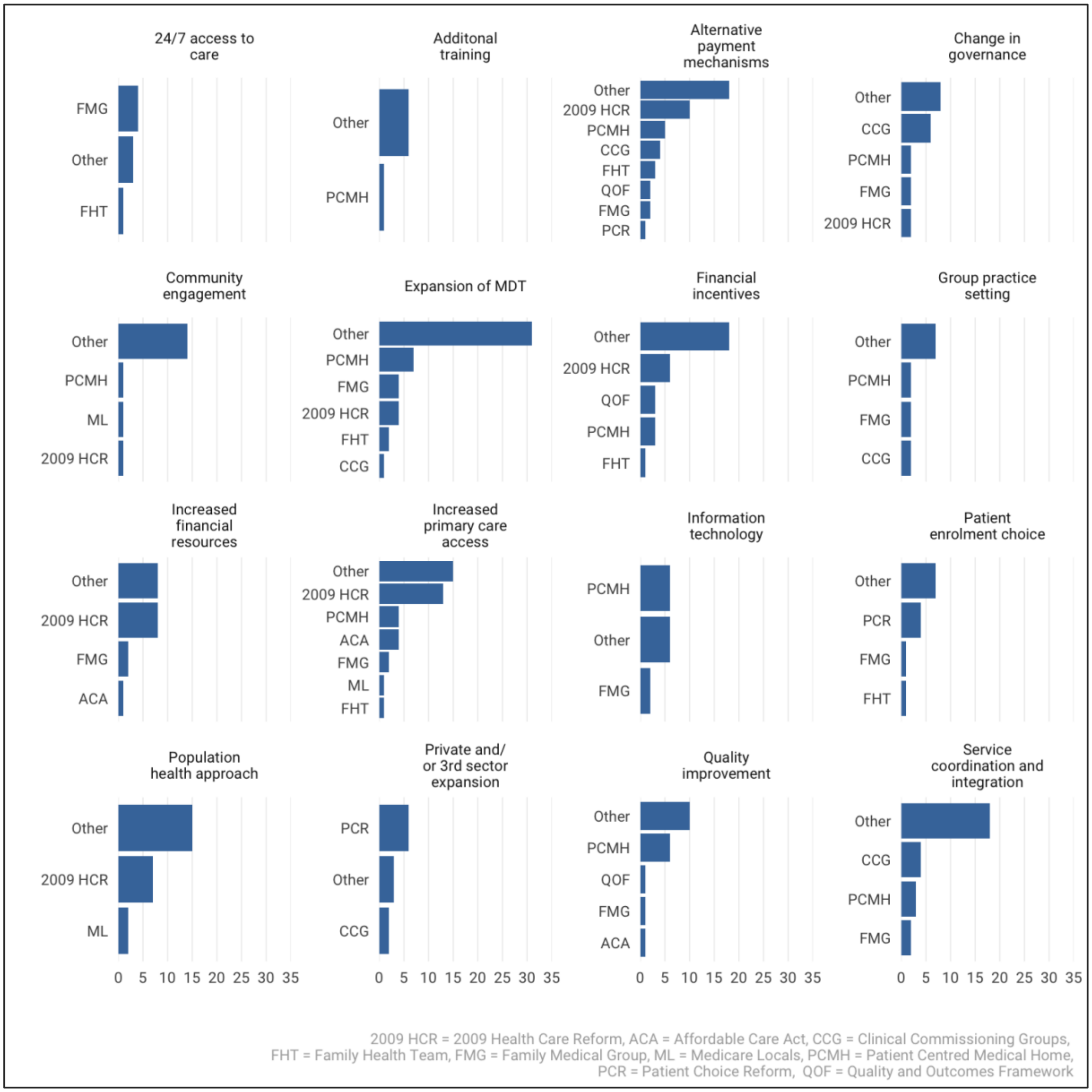

Figure S4: Count of outcome measures in included studies, by policy

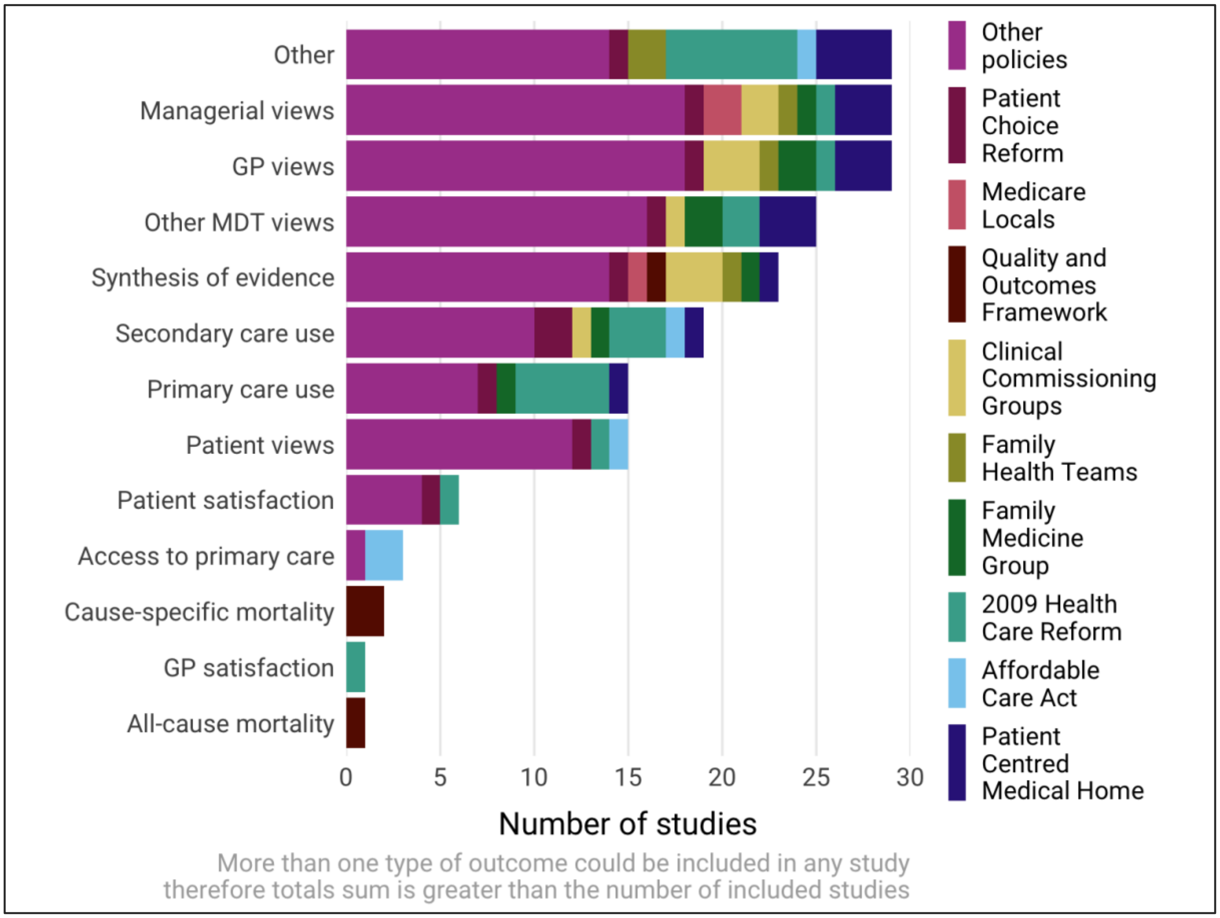

Figure S5: Outcome measures in included studies, by methodology

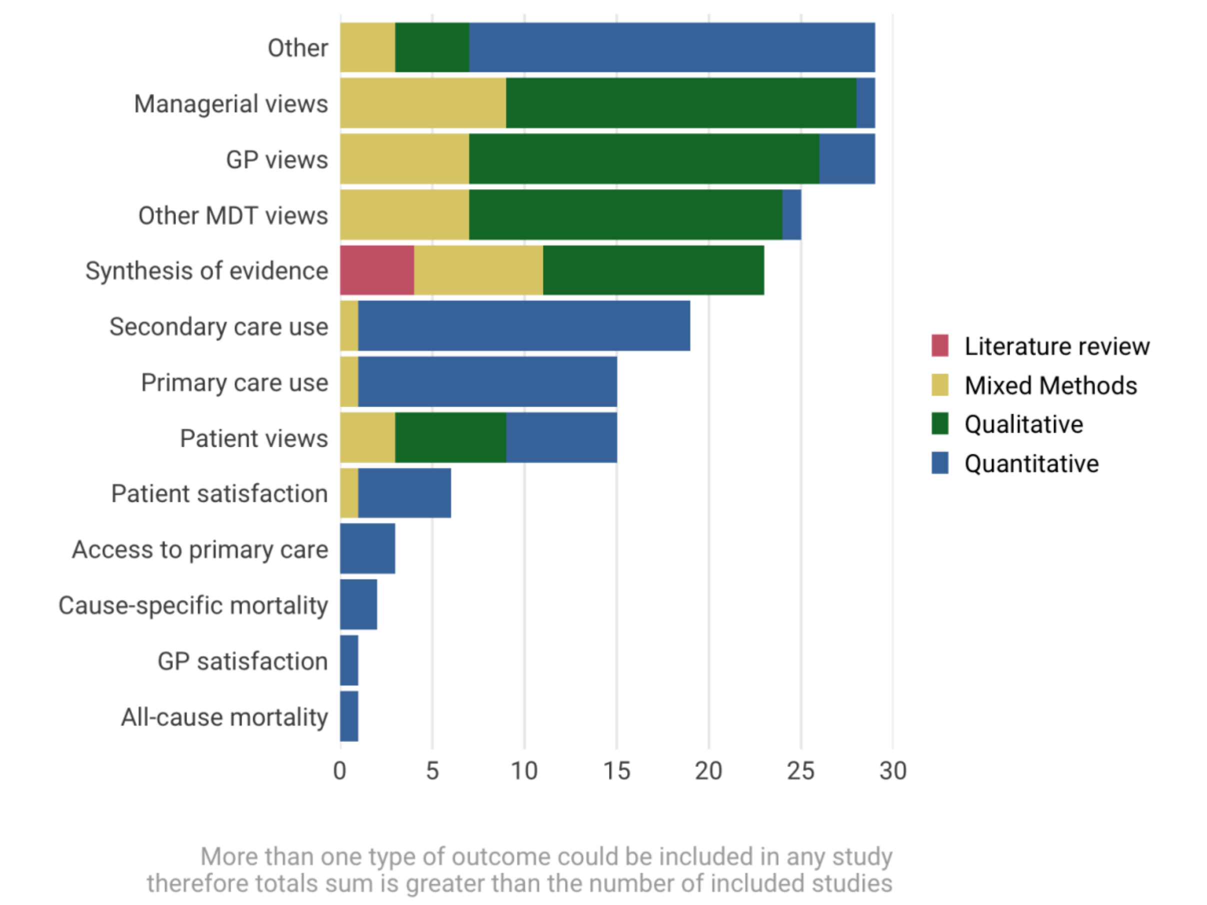

Figure S6: Types of health inequalities measured (n = 37)

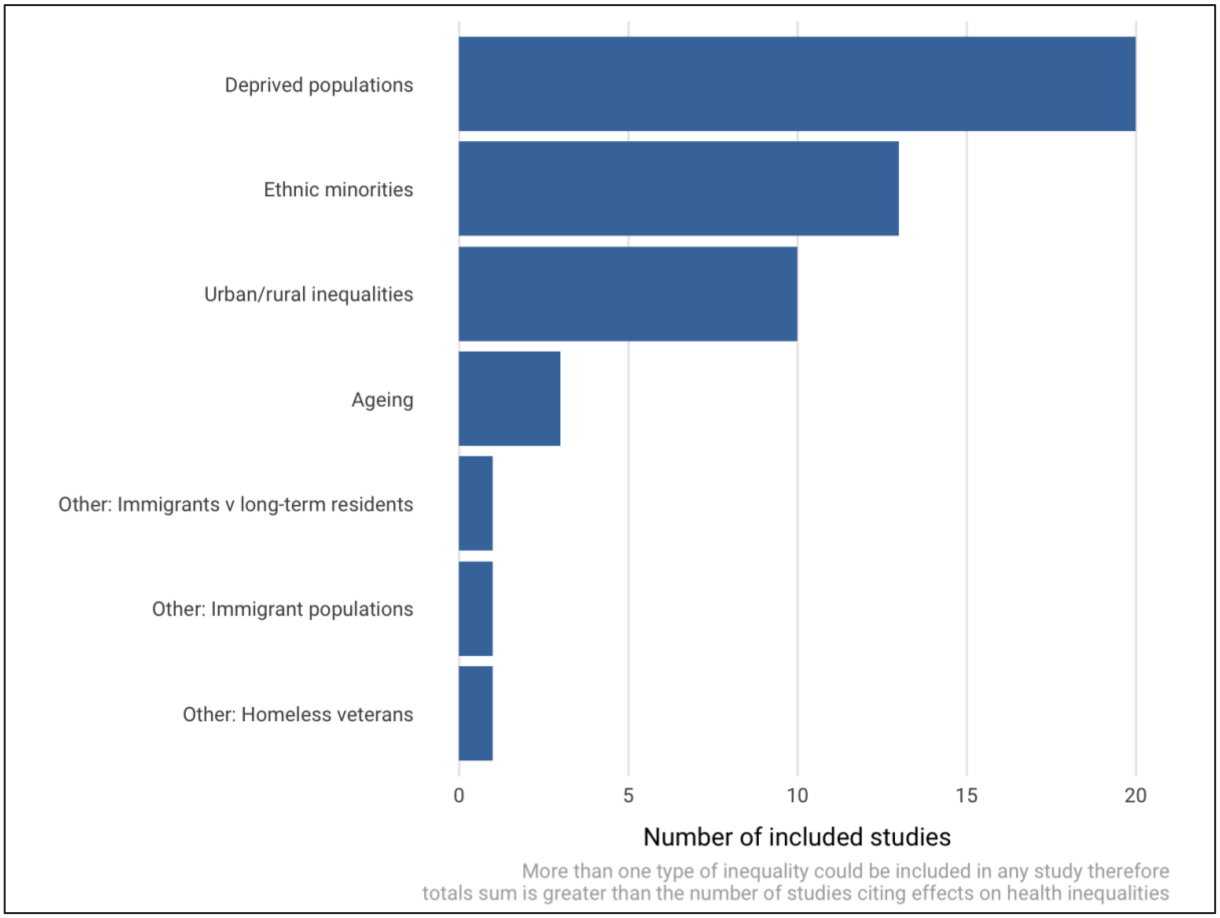

Figure S7: Studies citing specific barriers or facilitators to PCT (n = 41), by methodology and country

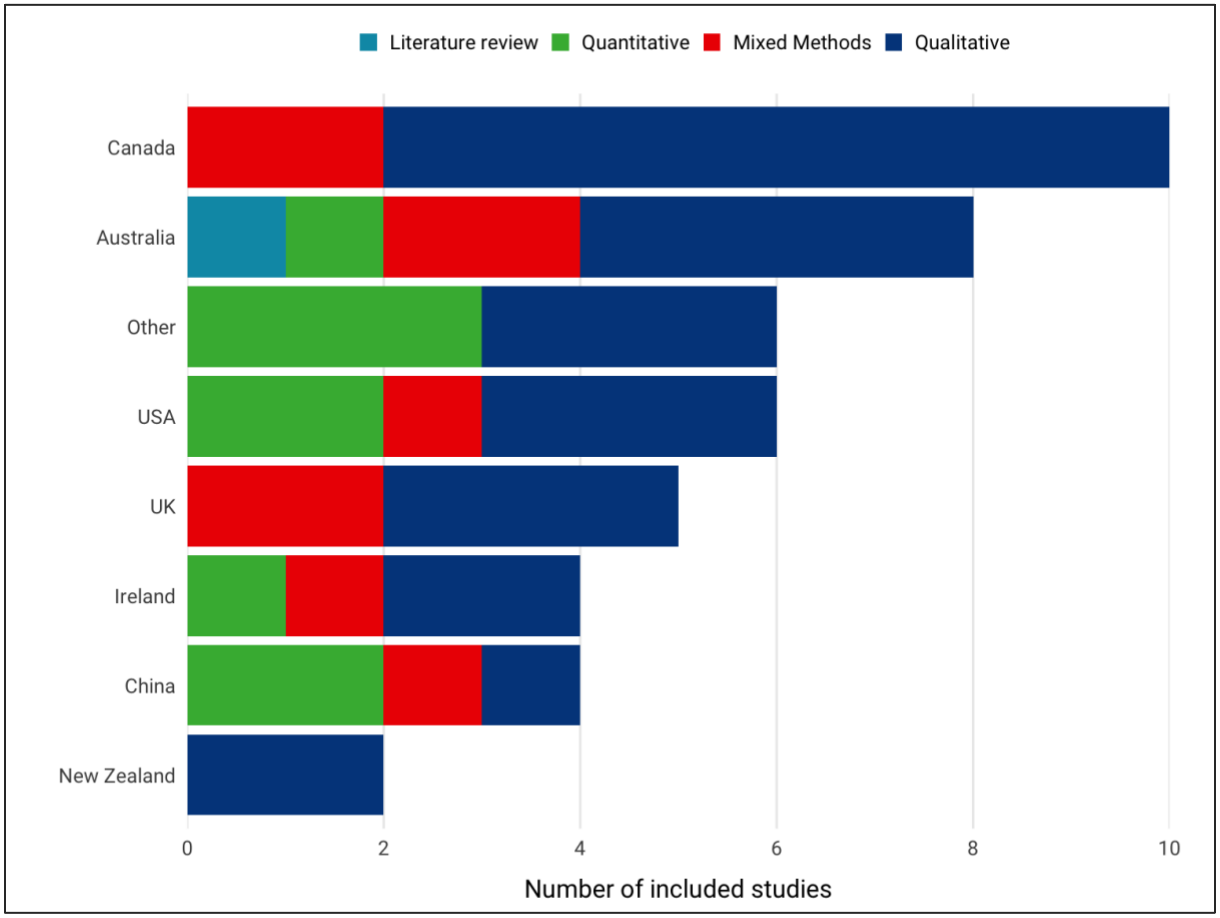

Figure S8: Included studies by year

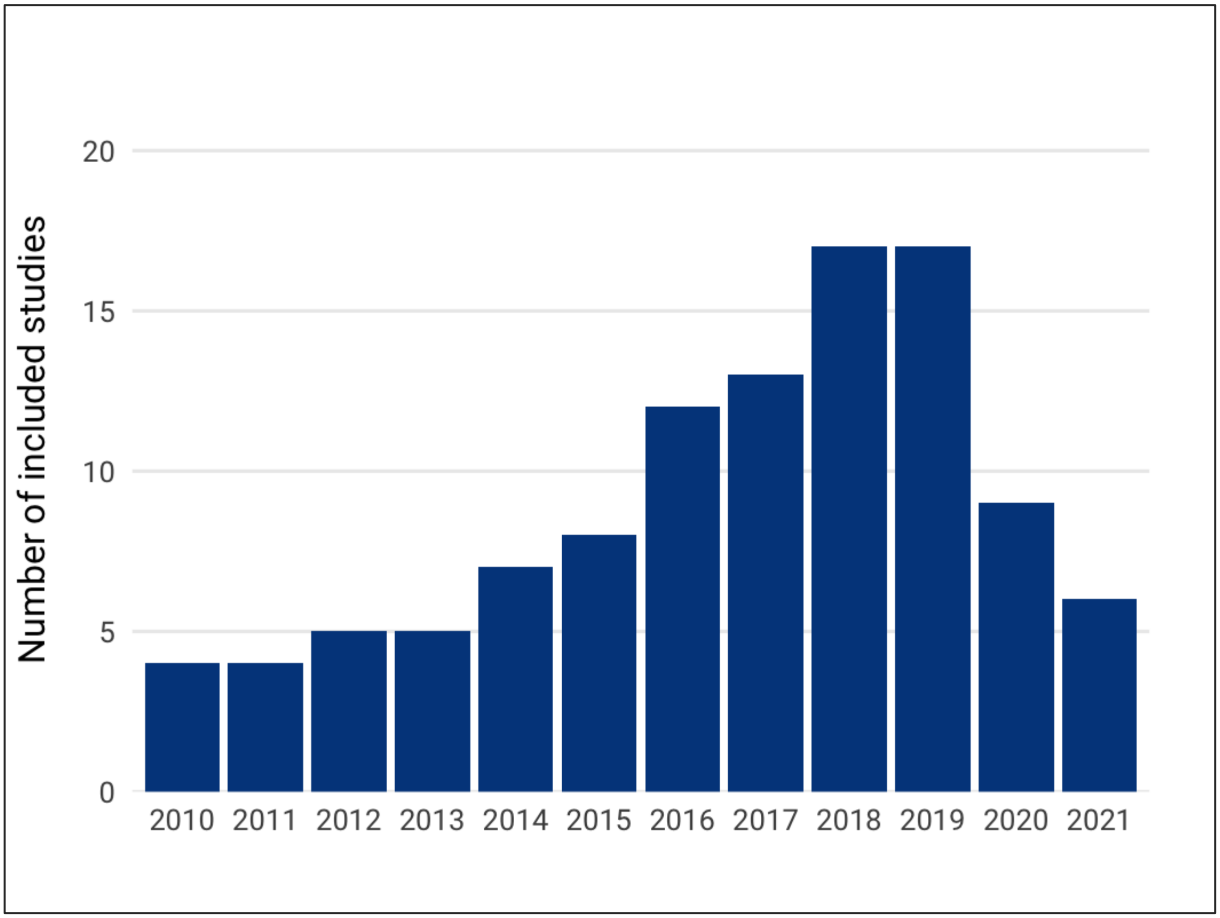

Supplement: Supplementary file 1 — Additional file 1: Table S1. PRISMA-ScR Checklist. Table S2. MEDLINE Search terms. Table S3. Characteristics of included studies. Fig. S1. Included studies by country and methodology. Fig. S2. Count of types of primary care transformation in included studies, by policy. Fig. S3: Count of types of primary care transformation in included studies, by policy (alt). Fig. S4. Count of outcome measures in included studies, by policy. Fig. S5. Outcome measures in included studies, by methodology. Fig. S6. Types of health inequalities measured (n=37). Fig. S7. Studies citing barriers and facilitators to PCT (n=41), by methodology and country. Fig. S8. Included studies by year [file 12916_2023_3033_MOESM1_ESM.pdf]
